# Siah2 integrates mitogenic and extracellular matrix signals linking neuronal progenitor ciliogenesis with germinal zone occupancy

**DOI:** 10.1038/s41467-020-19063-7

**Published:** 2020-10-20

**Authors:** Taren Ong, Niraj Trivedi, Randall Wakefield, Sharon Frase, David J. Solecki

**Affiliations:** 1 Department of Developmental Neurobiology, St. Jude Children’s Research Hospital, Memphis, TN 38105 USA; 2 Cell and Tissue Imaging Center-EM, St. Jude Children’s Research Hospital, Memphis, TN 38105 USA

**Keywords:** Cell migration, Ciliogenesis, Differentiation, Neurogenesis

## Abstract

Evidence is lacking as to how developing neurons integrate mitogenic signals with microenvironment cues to control proliferation and differentiation. We determine that the Siah2 E3 ubiquitin ligase functions in a coincidence detection circuit linking responses to the Shh mitogen and the extracellular matrix to control cerebellar granule neurons (CGN) GZ occupancy. We show that Shh signaling maintains Siah2 expression in CGN progenitors (GNPs) in a Ras/Mapk-dependent manner. Siah2 supports ciliogenesis in a feed-forward fashion by restraining cilium disassembly. Efforts to identify sources of the Ras/Mapk signaling led us to discover that GNPs respond to laminin, but not vitronectin, in the GZ microenvironment via integrin β1 receptors, which engages the Ras/Mapk cascade with Shh, and that this niche interaction is essential for promoting GNP ciliogenesis. As GNPs leave the GZ, differentiation is driven by changing extracellular cues that diminish Siah2-activity leading to primary cilia shortening and attenuation of the mitogenic response.

## Abstract (web summary)

In neural development, progenitors transition from a proliferative to a differentiated state. Here, the authors show that cerebellar granule neurons retract primary cilia as they exit their proliferative niche upon decreased ECM engagement, enabling radial migration due to loss of Shh sensitivity.

## Introduction

During neural development, neuronal precursors within germinal zone (GZ) niches simultaneously balance proliferation with the onset of differentiation by responding to morphogens or extracellular matrix (ECM) signaling molecules that have a mitogenic function. A seamless transition of neural precursors from a proliferative state to a terminally differentiated, migratory state underlies the competence of newborn neurons to leave the mitogen-rich GZ to populate the lamina, where these cells will connect with their targets in nascent neuronal circuits^1^. Despite recent progress in understanding the transcriptional cascades and cell-cycle regulatory mechanisms governing the period and output of progenitor proliferation events, relatively little is known about how progenitors process various morphogen and ECM stimuli to integrate responsiveness to mitogens with the onset of GZ exit or subsequent migration.

With its well-defined GZ niche, the cerebellar granule neuron (CGN) is an excellent model with which to study the impact of differentiation transitions on the integration of mitogen responsiveness to GZ exit. Granule neuron progenitors (GNPs) undergo proliferation within the external germinal layer (EGL) and, upon differentiating into CGNs, they migrate radially along the Bergmann glial scaffold, passing through a layer of Purkinje neurons before arriving at their final positions in the internal granule layer^2^. Two cell types provide local and long-range mitogenic signals to GNPs. Pial fibroblasts produce an ECM-rich basement membrane with which GNPs interact via integrin receptors to maintain their proliferation^3–6^. Purkinje neurons produce the ligand sonic hedgehog (Shh), a potent mitogen for proliferative GNPs in the outer EGL (oEGL)^7^. This pathway is frequently deregulated to transform GNPs in a distinct subgroup of cerebellar medulloblastomas (Shh-MBs)^8–10^. The transduction machinery for Shh is localized to the primary cilium, a microtubule-based organelle that is required for the response of GNPs to Shh and, ultimately, for proper cerebellar development^11^. Cilium-localized Patched (Ptch) receptor, the negative regulator of the hedgehog pathway, acts as a repressor of Smoothened (Smo), a G-protein–coupled receptor that is the principal activator of the hedgehog pathway^12^. Binding of the ligand Shh to Ptch relieves its repression of Smo^13^, which in turn triggers a series of downstream events that converge into the stabilization and nuclear translocation of the Gli transcription factors^14^. Importantly, Shh signaling has ceased by the time differentiated CGNs exit the EGL to undergo radial migration, and despite migrating against a gradient of Shh, the CGNs remain insensitive to the ligand. How developing GNPs turn the Shh pathway on or off and how their responsiveness is modified by local cues, such as pia-derived ECM, is unclear and is a key to understanding how normal and transformed GNPs expand during cerebellar development.

CGNs also have a well-characterized cell-intrinsic circuitry controlling GZ exit^15–20^. Prominent among these GZ-exit regulators is the seven in absentia 2 (Siah2) E3 ubiquitin ligase. This is an evolutionarily conserved cell-fate regulator that is the most downstream component of the Ras GTPase/Map kinase (MAPK) signaling pathway in the specification of R7 cells in the *Drosophila* retina^21^. Our laboratory has shown that, during CGN differentiation, Siah2 modulates the acquisition of neuronal polarity and the actin–microtubule interactions necessary for radial migration^16,20^. During the peak stages of progenitor cell expansion, Siah2 expression is high in GNPs, but it is extinguished in CGNs as they exit the EGL. Gain- or loss-of-function studies have shown that Siah2 is necessary and sufficient to maintain GZ occupancy, ultimately by targeting the Pard3 polarity protein and Drebrin (Dbn), the microtubule–actin cross-linking protein, for ubiquitin proteasome degradation. As Pard3 and Dbn act in CGNs to promote radial migration, the relief of Siah2-target inhibition in neuronal differentiation represents a form of integration for cell biological activities linked to GZ exit, positioning Siah2 as a mechanistic entry point through which to explore how GNPs maintain position in their germinal niche. Despite the central role of Siah2 in regulating GNP GZ exit, it is still unclear how extrinsic morphogens and signaling cascades regulating GNP differentiation affect the cell-intrinsic GZ circuity controlled by Siah2 activity or whether Siah2 actively functions to maintain the GNP state.

In this study, we find that, similar to invertebrate Siah orthologues, mouse Siah2 appears to function downstream of Ras/MAPK in CGN GZ exit. We uncover a surprising link between mitogen signaling and the CGN GZ-exit machinery, as Shh signaling maintains Siah2 expression in proliferative GNPs. We show that the perduring EGL that results from deregulated Shh signaling in GNPs can be rescued by blocking Ras/MAPK and Siah2. We serendipitously discover that CGNs achieve this effect by diminishing their primary cilia and thereby becoming insensitive to Shh, thus enabling them to migrate radially. Interestingly, Ras/MAPK and Siah2 activity are required to maintain primary ciliogenesis in dividing GNPs in part through Siah2 targeted degradation of the Pard3, Dbn, and Pitchfork proteins. Importantly, we show that the microenvironment surrounding the oEGL, which contains laminin substrates, promotes primary ciliogenesis in proliferative GNPs in an integrin receptor-dependent manner. In our new GZ-exit model, as differentiating GNPs exit their laminin-rich niche, they lose the supportive signals that are required for them to maintain their primary cilia. Siah2 is central to this model because GNPs employ this ubiquitin ligase to maintain GZ occupancy and sensitivity to Shh signaling in a process that is integrated with the engagement of ECM components in their niche.

## Results

### Ras–Raf–MAPK pathway controls GZ exit by regulating Siah2

**Fig. 1:**
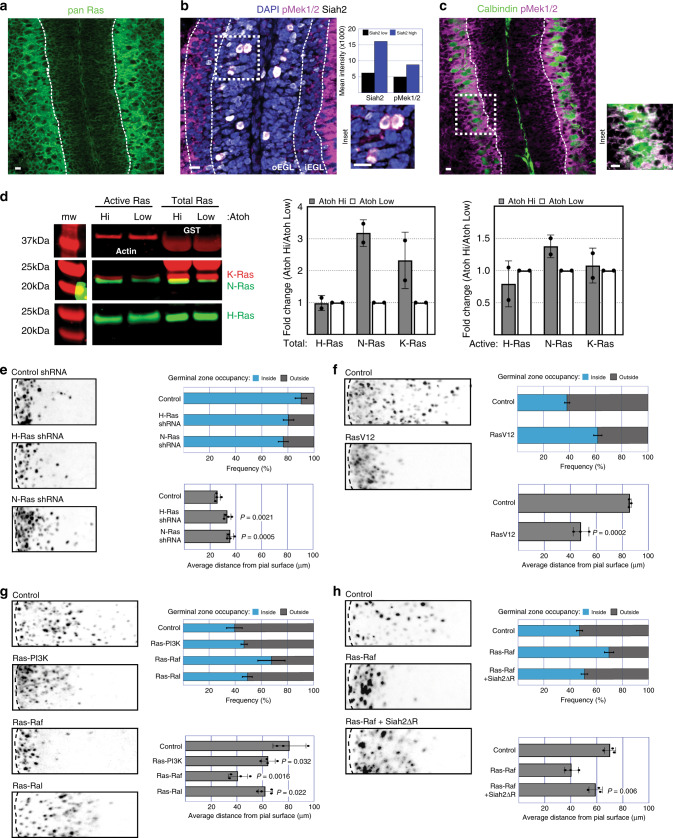
Ras/Mapk controls GZ exit in a Siah2-dependent manner. Representative images of immunofluorescent assessment of **a** Ras, **b** Siah2, **c** phospho-Mek1/2 (pMek1/2), and calbindin in an internal folium of a P7 cerebellum. Assessment performed on *n* = 3 independent P7 WT cerebella. Dotted lines demarcate the two opposing EGLs of an internal folium. Dotted-lined square boxes in **b** and **c** represent insets. Quantification in **b** shows the mean intensity of pMek1/2 in Siah2 high or low expressing cells of the single confocal image. Segmentation strategy provided in Source Data. **d** Western blot showing the expression of the active and total Ras isoforms in whole cell lysates of FACS-sorted GNPs (Atoh Hi) and newly differentiated CGNs (Atoh Low). Western blot quantification represents *n* = 2 independent experiments of pooled populations of at least 30–40 P6 and P7 cerebella. Full scans of blots available in Fig. S1. **e**–**g** Ex vivo cerebellar pulse-chase assays, examining the effect of **c** Ras knockdown (four independent experiments); **d** RasV12 overexpression (three independent experiments); **e** activation of Ras downstream effectors Ras–PI3K (RasV12Y40C), Ras–Raf (RasV12T35S), or Ras–Ral (RasV12E37G), (four independent experiments); and **f** rescue of Ras–Raf activation by Siah2 (three independent experiments), in P7 GNPs. Quantifications show percent frequency distribution within and outside the EGL (top panel) and the mean migration distances (lower panel), which accounts for at least *n* > 5000 individual cells counted in every condition. Horizontal length of representative images represents radial distance of 200 μm (**c**), 300 μm (**d–f**) from cerebella pial. *P* values represent unpaired one-tailed student’s *t* test performed compared to control (**e–g**) or Ras–Raf (**h**). Scale bar = 10 μm. Error bars represents mean ± SD.

Intrigued by the reported connection between the Ras/MAPK signaling pathway and Siah E3 ubiquitin ligases^22–24^, we were curious as to whether this relationship was conserved in GNP neurogenesis and GZ exit. We first determined the expression of Ras and phospho-Mek1/2 (pMek1/2) in the developing cerebellum. Immunofluorescence staining for pan Ras and pMek1/2 in combination with Siah2, as a marker for the oEGL, was performed on P7 cerebella from wild-type (WT) mice. Within the EGL, the expression of Ras (Fig. 1a) was uniform throughout, whereas the expression of pMek1/2 (Fig. 1b), a functional readout for active Ras/MAPK signaling, coincided with GNPs having high levels of Siah2 expression as shown in the mean intensity measurements of pMek1/2 levels in Siah2 high vs. low cells. The high level of pMek1/2 staining in the molecular layer seems to originate from the Purkinje neurons as judged by their co-localization with calbindin (Fig. 1c). As Siah2 expression in GNPs decreases as they differentiate into CGNs, we wanted to know whether Ras expression followed a similar trend. Ras is a GTPase that cycles between active and inactive states and is capable of signaling to its effectors only when it is in the active state^25^. Using the Atoh1-GFP reporter mice^26^ where GFP is expressed under the GNP specific promoter, Atoh1, we sorted cells based on high or low expressions of GFP which corresponds to GNPs or newly differentiated CGNs, respectively. Whole cell lysates were collected from these populations and subjected to western blotting plus active Ras pull-down for the three main Ras isoforms—H, N, and K (Fig. 1d). While total expression H-Ras was unchanged by differentiation status, N- and K-Ras expression was elevated in GNPs. Moreover, we found that N-Ras activity, the dominant isoform found in neuronal cells, was significantly higher in GNPs.

Given the evidence that Ras activity diminishes in a temporal window where GNPs mature into CGNs, we wanted to know whether Ras played a role in regulating GZ exit and therefore used a knockdown or gain-of-function approach to study Ras in GNPs. By using the ex vivo cerebellar pulse-chase assay developed by our laboratory (see “Materials and methods”), we can specifically label GNPs and track their migration^16,19,27^. The EGLs of P7 WT cerebella were electroporated with miR-30-based shRNAs against H-Ras or N-Ras or against *Renilla* luciferase as a control (Fig. 1e). Twenty-four hours post electroporation (hpe), the control cells remained within the EGL, as can be seen by the appearance of a tight band of cells at the surface of the cerebellum. In the H-Ras- or N-Ras-silenced EGLs, however, the cells appeared to begin migrating out of the EGL, with N-Ras silencing having the greatest effect in spurring GZ exit. In contrast, the overexpression of a constitutively active mutant of H-Ras, H-Ras (V12)^28^, in the EGL (Fig. 1f) completely blocked GZ exit at 48 hpe.

Ras triggers the activation of several downstream effector pathways, including the PI3K, Raf–MAPK, and Ral signaling cascades^29^. To determine the pathway responsible for controlling GZ exit in GNPs, we electroporated P7 WT cerebella with the constitutively active mutant forms of H-Ras(RasV12) that primarily activate the PI3K (Y40C), Raf–MAPK (T35S), or Ral (E37G) pathways^29^ (Fig. 1g). By 48 hpe, most of the control cells had exited the GZ and migrated deep into the cerebellum. The expression of the PI3K- and Ral-activating Ras mutants dampened GZ exit only mildly, whereas the Raf-activating Ras mutant exhibited a greater inhibitory effect that was similar in magnitude to that of Ras V12 (Fig. 1f). This suggests that GZ exit controlled by Ras is primarily mediated through the Raf–MAPK pathway. We hypothesized that if Siah2 functioned downstream of the Ras–Raf pathway, then inhibiting its function in the event of constitutive activation of the Ras–Raf pathway should rescue GZ exit. Indeed, by overexpressing a dominant-negative form of Siah2 with a truncated RING domain^30^ concurrent with the Raf-activating Ras mutant, we could restore GZ exit (Fig. 1h). The results of these epistasis experiments suggest that the Ras–MAPK pathway controls GZ exit through Siah2.

### Shh regulates Siah2 in a Ras–Raf–MAPK-dependent manner

**Fig. 2:**
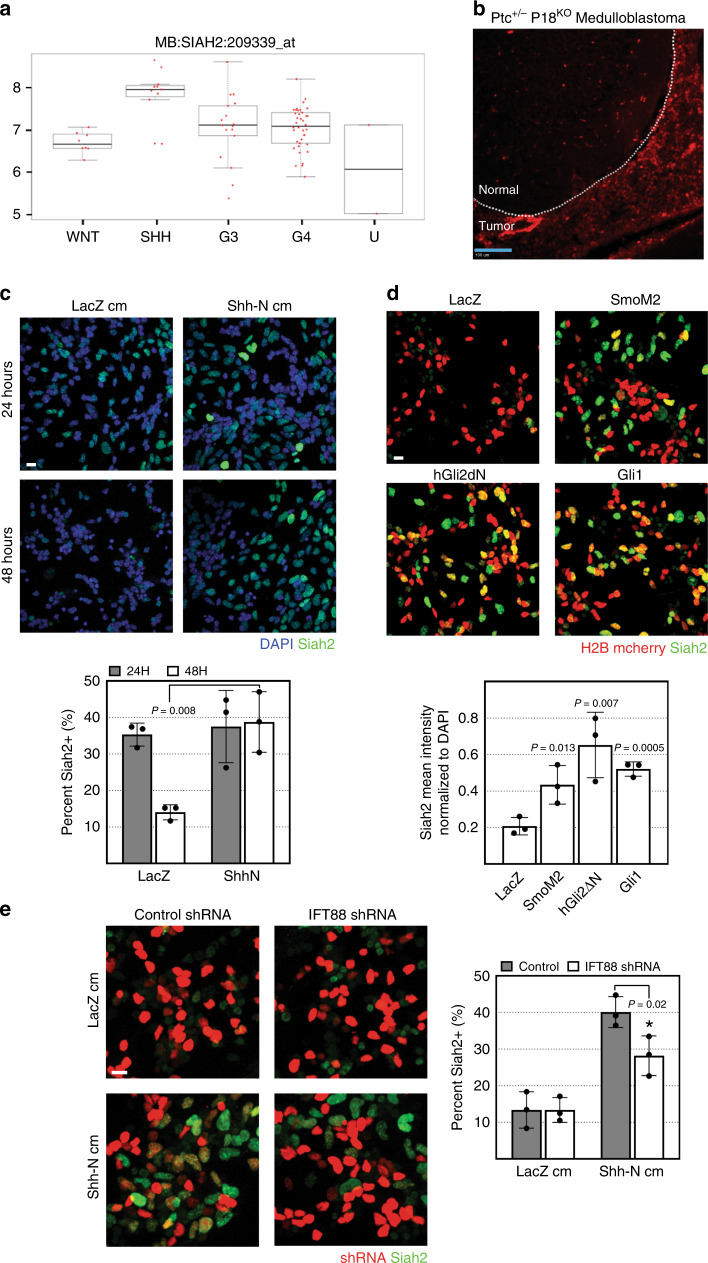
Shh maintains Siah2 expression in a Ras/Mapk-dependent manner. **a** Siah2 transcript expression in the different subgroups of human medulloblastomas. U uncategorized (the subset of tumors that do not belong to any of the four subgroups). Data obtained from the Pediatric Cancer Genome Project (https://pecan.stjude.cloud/proteinpaint/SIAH2). Boxplots represents the median, interquartile range, maximum and mininum. **b** Representative immunofluorescent staining of Siah2 on *n* = 3 independent mouse *Ptc*^*+/−*^ *P18*^*KO*^ medulloblastoma tumors. Dotted lines demarcate the boundary between normal and transformed tissue. Light blue scale bar = 100 μm. **c**–**e** Isolated GNPs from P7 WT cerebella were cultured on Matrigel-coated glass as indicated. cm conditioned medium. **c** Cells were fixed after 24 and 48 h followed by Siah2 immunofluorescence staining. Quantification shows the mean percentage of Siah2-positive cells. **d**, **e** Cells were co-nucleofected with the nuclear marker H2B-mCherry and the indicated constructs and cultured for 48 h followed by fixation and immunofluorescence for Siah2. Siah2 expression was measured using SlideBook. Quantification shows mean intensity measurements (**d**) or percent positive (**e**). All quantifications **c**–**e** represent 3 biological replicates with *n* = 18 confocal image fields analyzed. *P* values represent unpaired one-tailed student’s *t* test performed when compared to LacZ (**d**) or as indicated. Scale bar = 10 μm. Error bars represents mean ± SD.

Ras–MAPK generally functions with mitogen signaling receptors to regulate cellular proliferation^31^. Having determined that Siah2 functions downstream of the Ras–MAPK pathway in GNP GZ exit, we next wished to identify an appropriate mitogen that interfaced with Ras/MAPK/Siah2 in our system. We focused on the Shh morphogen because the prototypical mitogens that signal through receptor tyrosine kinase receptors to stimulate Ras/MAPK activity (e.g., EGF, IGF, or Fgf) are very poor GNP mitogens, whereas Shh is an order of magnitude more potent as a GNP mitogen than any other morphogen tested to date^10,32^. Our decision was bolstered by data from the Pediatric Cancer Genome Project (PCGP), which revealed that in human MBs, a group of tumors that arise from GNPs^33^, the expression of Siah2 transcripts was highest in the Shh subgroup of the tumors (Fig. 2a). To extend the finding of Siah2 expression in a defined genetic context in which Shh signaling is genetically activated in GNPs, we performed  immunofluorescent staining for Siah2 on samples of Shh-driven MBs obtained from the Ptc^+/−^ P18^KO^ MB mouse model^34^ (Fig. 2b). The staining revealed that Siah2 was expressed within the tumor but not in neighboring untransformed cerebellar tissue. To further determine whether Shh signaling regulated Siah2 in untransformed progenitors of the CGN lineage, we grew isolated CGNs from P7 WT cerebella in culture in LacZ- or Shh-N-conditioned medium (Fig. 2c). The expression of Siah2 remained unchanged after 24 h. However, at 48 h, the control cells cultured in LacZ-conditioned medium, but not those cultured in Shh-N-conditioned medium, had downregulated Siah2, suggesting that Shh signaling could maintain Siah2 expression. We confirmed this finding by overexpressing the following proteins that activate the Shh pathway in GNPs: Smoothened-M2 (SmoM2)^35^, a constitutive active mutant of Smoothened; Gli1^36^, the transcription factor upregulated in response to Shh pathway activation; and Gli2ΔN^37^, a truncated form of Gli2 that acts as a transcription activator; along with LacZ as a control (Fig. 2d). All but the LacZ-encoding construct could drive Siah2 expression. We tested whether Shh signaling was required for Siah2 expression by using a cilium-deletion approach in which we silenced Intraflagellar transport protein 88 (Ift88). Shh signal transduction obligately requires a primary cilium, and its depletion via Ift88 or Kif3a loss of function renders GNPs insensitive to Shh signaling^38–40^. Control cells showed a strong increase in Siah2 expression in response to Shh-N stimulation, but this increase was dampened in Ift88-knockdown cells (Fig. 2e). We also confirmed that Ift88 knockdown decreased the number of primary ciliated cells in culture (Supplementary Fig. 2A), while application of small molecule inhibitors of Gli transcription factors also inhibited Shh-N driven Siah2 expression (Supplementary Fig. 2B). Finally, we assessed Siah2 transcript level in CGNs stimulated with SAG for 24 h. Shh signaling led to a robust increase in Gli1 mRNA, a canonical target of the cascade, at 24 h of stimulation, a time point where Siah2 mRNA level increased about 50% (Supplementary Fig. 2C). Taken together, these results show that Shh signaling is necessary and sufficient to maintain Siah2 expression in GNPs. Shh maintainence of Siah2 expression has some canonical Shh signaling features, i.e., requires cilia and Gli transcription factors, however these assays cannot rule out additional post-transcriptional or noncanonical regulation of Siah2.

Our epistasis experiments (Fig. 1) showed that Siah2 functions downstream of the Ras–MAPK cascade in controlling GZ exit. To determine whether the maintenance of Siah2 expression by Shh signaling required the Ras–MAPK cascade, we first used a pharmacologic inhibitor approach. We stimulated isolated CGNs from P7 WT cerebella with SAG, a small-molecule agonist of the Shh pathway, for 48 h in the presence of vehicle control or one of the following inhibitors of the Ras–MAPK pathway: farnesyl thiosalicylic acid (FTA), a Ras inhibitor; sorafenib (Sor), a Raf inhibitor; trametinib (Tra), a Mek inhibitor; or FR180204 (FR), an Erk inhibitor (Supplementary Fig. 3). Pharmacologic inhibition of any component of the Ras–MAPK cascade in the presence of SAG decreased Siah2 expression.

**Fig. 3:**
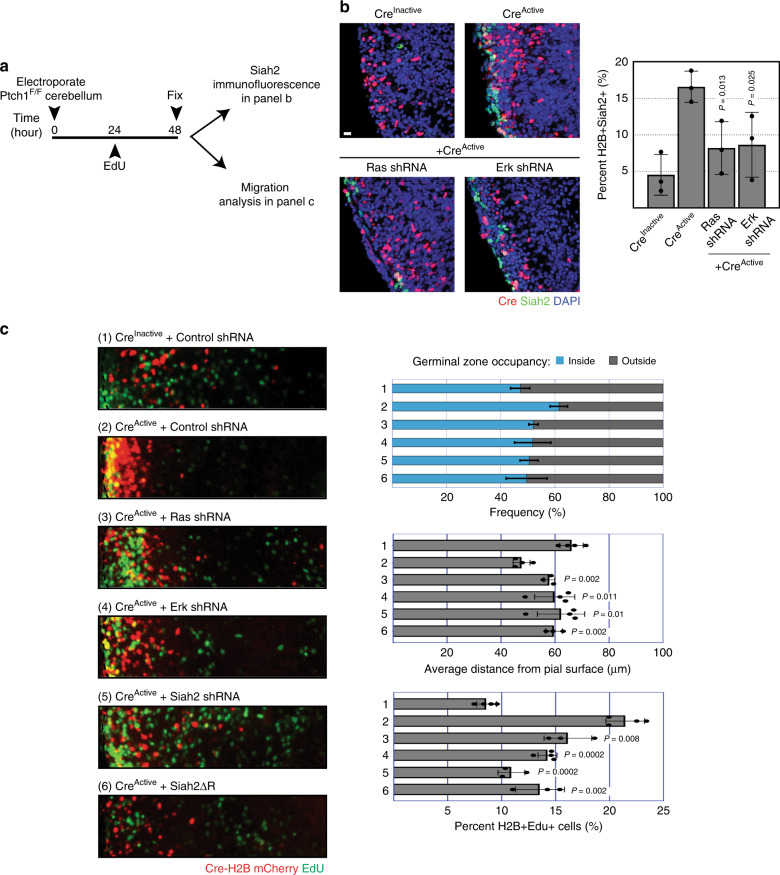
Shh signaling blocks GZ exit in a Ras/Mapk and Siah2-dependent manner. **a** Ex vivo cerebellar pulse-chase experiment schema using P7 *Ptch1*^*Flox/Flox*^ (Ptc^F/F^) cerebella for panels **b** and **c**. **b** Post fixation, the cerebellar slices were processed for immunofluorescent staining of Siah2. Quantification shows mean Siah2-positive cells in H2B-mCherry electroporated cells (Cre^Inactive^ + Erk shRNA, *n* = 28; all other conditions, *n* = 31, confocal image fields sampled from 3 independent experiments on 3 different cerebella). **c** Post fixation, the cerebellar slices were stained for EdU using the Invitrogen Click-it Edu staining kit according to the manufacturer’s instructions. Quantification of 4 independent experiments show the mean frequency distribution within and outside the EGL (top panel), the average migration distance (middle panel), and the percentage of electroporated cells that were EdU positive (lower panel), which accounts for at least *n* > 8000 individual cells counted in every condition. Horizontal length of representative images represent radial distance of 300 μm from cerebella pial. *P* values represents unpaired one-tailed student’s *t* test when compared to Cre^Active^ + Control shRNA. Scale bar = 10 μm. Error bars represents mean ± SD.

We further tested the dependency of Ras–MAPK signaling on Shh maintenance of Siah2 expression by using our ex vivo cerebellar slice system. The EGLs of P7 *Ptch1*^*Flox/Flox*^ cerebella were electroporated with plasmids encoding codon-optimized Cre recombinase or its inactive mutant to create cohorts of GNPs with and without strong genetic activation of the Shh signaling cascade; Ptch1 loss of function relieves its restraint on Smo function, leading to constitutive activation of the Shh pathway. Consistent with the results of our in vitro SAG treatment, immunofluorescent staining for Siah2 revealed that Siah2 expression was increased in *Ptch1*^*Flox/Flox*^ GNPs expressing Cre recombinase (Fig. 3b). Importantly, Siah2 expression was decreased to control levels by knocking down H- and N-Ras or Erk, showing that Shh maintenance of Siah2 expression in GNPs residing within the EGL niche requires the Ras–MAPK cascade.

### Shh signaling delays GZ exit through Ras–Mapk and Siah2

An overt phenotype of sustained Shh signaling in GNPs is a perduring EGL due to a delay in GZ exit. This has been demonstrated in various Shh-MB mouse models^41–43^. Although Shh signaling maintains GNPs in a proliferative and undifferentiated state, the Shh-regulated downstream mechanisms controlling GZ exit remain unclear. Given that Siah2 blocks GZ exit in GNPs by preventing their differentiation and polarization, we postulated that Shh signaling blocked GZ exit by maintaining Siah2 expression in a Ras–MAPK-dependent manner. To test this using the ex vivo migration assay, we electroporated EGLs of P7 *Ptch1*^Flox/Flox^ cerebella with plasmids encoding codon-optimized Cre recombinase or its inactive mutant and maintained the cerebella slices in ex vivo culture for 48 h (Fig. 3c). EdU was added in the final 24 h of culture to assess proliferation. GZ exit in GNPs expressing the catalytically inactive mutant Cre recombinase proceeded normally with low incorporation of EdU, demonstrating that cell-cycle exit and differentiation were normal in GNPs with two *Ptch1* alleles. In contrast, GZ exit was retarded in GNPs expressing active Cre recombinase, and this was coupled with an increase in EdU incorporation. This finding was expected, because sustained Shh signaling due to the loss of both *Ptch1* alleles should maintain GNPs in a proliferative state. The GZ-exit phenotype due to the loss of *Ptch1* can be partially rescued by knocking down H- and N-Ras or by co-expressing the dominant-negative form of Siah2, Siah2ΔR, and it can be fully rescued by knocking down Erk1 and Erk2 or Siah2 (shRNA validations are shown in Supplementary Fig. 3B). Proliferation was decreased under all rescue conditions. These data are consistent with the GZ-exit phenotypes and show that sustained Shh signaling blocks GZ exit in GNPs by maintaining Siah2 expression in a Ras–MAPK-dependent manner.

### Differentiated CGNs retract their primary cilia

One potential interpretation of the requirement for Ras/MAPK signaling in Shh maintenance of Siah2 expression was that Ras/MAPK was downstream of the Shh signaling cascade. To test this, CGNs were stimulated with Shh-N conditioned medium for 15 or 30 min and whole cell lysates were subjected to active Ras pull-down followed by immunoblotting for Ras isoforms (Supplementary Fig. 4). We discovered that Shh-N stimulation activated the three main Ras isoforms (mechanism of which will be discussed in the final section). Concomitantly, we discovered serendipitously that Ras/MAPK and Siah2 co-operate to regulate GNP ciliation levels (discussed in the next section), the initial foundation of this line of investigation was a comprehensive analysis of ciliation in CGNs and GNPs. We found that GNPs placed in culture in the presence of SAG, the small-molecule agonist of the Shh pathway, maintained their primary cilia after 48 h in culture, whereas non-stimulated controls had shorter or no primary cilia (Supplementary Fig. 5). This suggests that proliferative GNPs maintain their primary cilia, whereas differentiated CGNs retract their primary cilia.

**Fig. 4:**
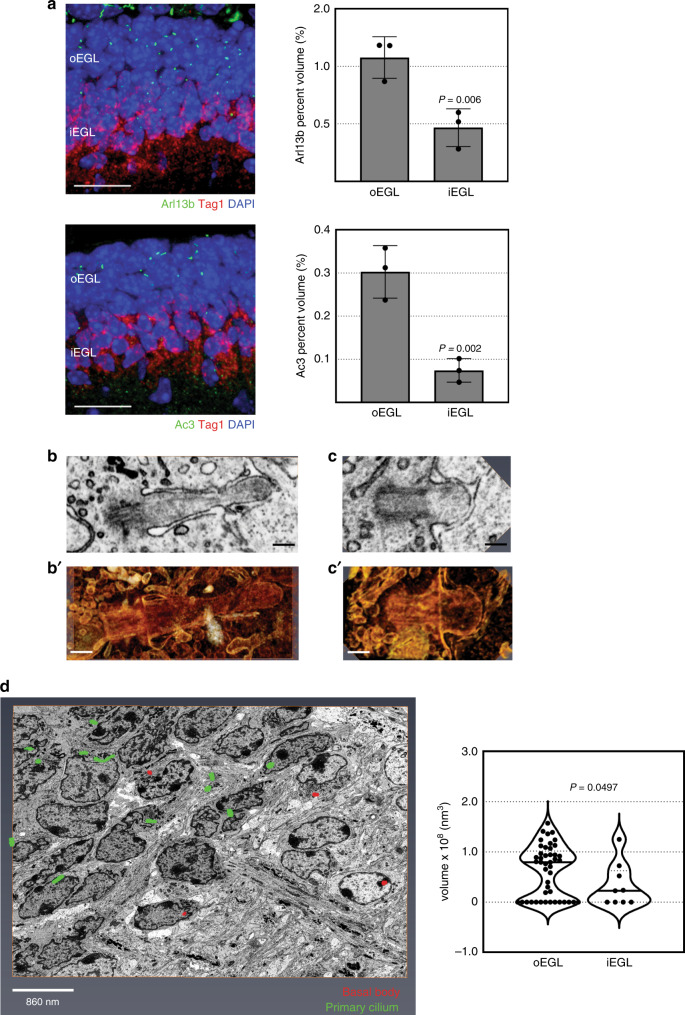
Differentiated CGNs retract their primary cilia. **a** Immunofluorescent co-staining for the primary cilium markers Arl13b or Ac3, and Tag1, the marker for newly differentiated CGNs, in 14 μm thick P7 cerebella cryo-sections. Confocal stacks (10 μm) were obtained for volumetric analyses of Arl13b or Ac3 using Amira. Quantification from 3 independent experiments, 3 independent cerebella, of *n* = 15 (Ac3) and *n* = 14 (Arl13b) confocal image stacks analyzed, shows the relative abundance of primary cilia within the oEGL and iEGL represented as the percentage volume within the inner and outer layers of the EGL. Scale bar = 10 μm. Representative FIB-SEM images of a single plane through the primary cilium of a GNP (**b**) and (**c**) CGN. **B’**, **C’** shows the corresponding 3D rendering. Black or white scale bars = 250 nm. **d** Representative maximum projected FIB-SEM image of the EGL in a P7 cerebellum. Primary cilium or basal body are segmented in green or red respectively. Quantification of ciliary volume (oEGL cilia *n* = 45; iEGL cilia *n* = 9) are represented in the Violin plot. FIB-SEM data **b**–**d** generated from *n* = 1 P7 WT cerebellum. *P* values in **a** derived from the unpaired one-tail student’s *t* test comparison between groups. *P* value in **d** derived from the Mann–Whitney test performed comparing the datapoints between groups. Violin plot represents median and distribution of datapoints. Error bars represents mean ± SD.

To further investigate our hypothesis, we used two approaches to examine the primary cilia in the developing cerebellum in vivo. First, we performed immunofluorescent staining on frozen P7 cerebellar sections from WT mice to detect the primary cilium markers ADP ribosylation factor-like GTPase 13B (Arl13b)^44^ and adenylate cyclase 3 (Ac3)^45^, along with co-staining for Tag1, which marks newly differentiated CGNs^46^ (Fig. 4a). We constructed a 3D rendering of the EGL based on the confocal images and performed a volumetric analysis of the primary cilia. Staining for both primary cilia markers showed that the primary cilia were more abundant in the oEGL than in the iEGL. To determine the changes in the axonemal volume of the primary cilia within the different layers of the EGL, we examined the EGLs of P7 cerebella by using 3D electron microscopy. The typical primary cilium of a GNP and its 3D-rendered image are shown in Fig. 4b, b′, respectively, and the primary cilium of a CGN and its 3D-rendered image are shown in Fig. 4c, c′, respectively. We manually segmented the primary cilia so as to encompass the basal body to the tip of the axoneme. An example of a 3D-rendered data set of a sampled region of the EGL is shown in Fig. 4d and Supplementary Video 1. Manual segmentation and volumetric analyses of the primary cilia, performed using Amira software, revealed that the primary cilia of cells within the oEGL are larger than those of cells in the iEGL. These data suggest that differentiated CGNs shorten their primary cilia to become insensitive to Shh and thereby downregulate this pathway to initiate GZ exit.

To determine whether ciliation status had any consequence for GNP GZ occupancy, we electroporated shRNA constructs against Ift88 and *Renilla* luciferase into the EGLs of P7 WT cerebella (Supplementary Fig. 6). After 24 h, we detected early GZ exit when we knocked down the primary cilia. The decreased EdU incorporation with Ift88 knockdown demonstrated that the primary cilia were efficiently knocked down, as proliferation is a direct response to Shh signaling in GNPs. Taken together, these data show that GNPs require their primary cilia in order to maintain GZ occupancy.

### Primary ciliogenesis and GZ occupancy require Mapk and Siah2

Given that knockdown of Ras or Siah2 induces early GZ exit, we wanted to know whether the Ras–MAPK pathway or Siah2 had a role in regulating the primary cilia. We nucleofected isolated CGNs from P7 WT cerebella with miR30-based shRNA constructs targeting H-Ras and N-Ras, Erk1 and Erk2, Siah2, or *Renilla* luciferase as a control, and we grew them in culture with or without Shh-N (Supplementary Fig. 7). After 48 h, the percentage of ciliated GNPs detected by Arl13b immunofluorescent staining was higher in Shh-N-treated cultures than in untreated control cultures. The percentage of ciliated cells was significantly decreased when Ras, Erk, or Siah2 was knocked down, showing that the maintenance of primary cilia in response to Shh requires Ras, Erk, and Siah2.

**Fig. 5:**
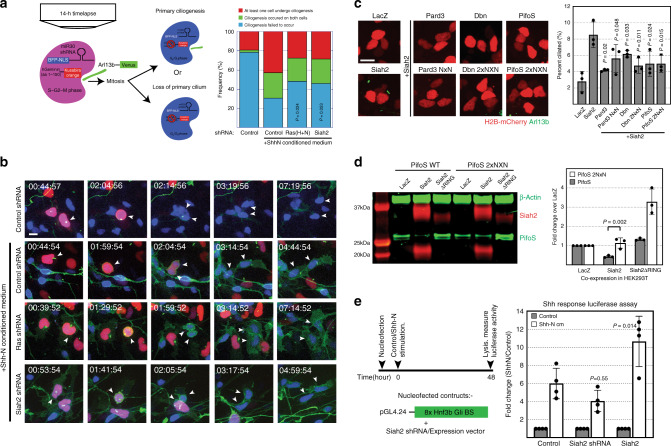
Siah2 supports primary ciliogenesis by inhibiting Pifo and Pard3. **a** Schema of the time-lapse ciliogenesis assay. Isolated P7 CGNs were nucleofected with a combination of the indicated expression vectors and cultured overnight in the presence or absence of Shh-N. Cells expressing all three vectors (BFP–NLS-tagged shRNA, Kusabira orange-tagged geminin, and Venus-tagged Arl13b) were selected, and time-lapse confocal imaging (7 min intervals) for 14 h was set up. **b** Representative frames of the process of primary ciliogenesis or lack thereof are as shown. The cells were scored according to whether ciliogenesis (1) observed on only 1 cell, (2) observed on both cells, or (3) fail to occur. Quantification (Control shRNA, *n* = 46; Control shRNA + Shh-N, *n* = 72; Ras(H + N) shRNA + Shh-N, *n* = 58; Siah2 shRNA + Shh-N, *n* = 59; independent cells analyzed) shows the frequency of the cells that fall within the 3 observations. Due to limited data points that fit the criteria, the result from this analysis was pooled from *n* = 9 independent experiments. *P* values derived from the *χ*^2^ test (2 degrees of freedom) comparing experimental groups to “Control shRNA + Shh-N conditioned medium”. **c** Isolated P7 CGNs were co-nucleofected with the indicated constructs and the bicistronic vector expressing Venus-tagged Arl13b and mCherry-tagged histone H2B and cultured in vitro for 48 h. Live-cell imaging was performed followed by quantification of at least *n* > 1000 cells in each condition from 3 independent experiments, for the proportion of nucleofected cells (H2B-mCherry+) that were ciliated. *P* values derived from an unpaired one-tail student’s *t* test comparing experimental groups to “Siah2”. **d** Western blot analysis of PifoS or PifoS 2NxN stability in the presence of Siah2. HA-tagged PifoS or PifoS 2N×N was co-transfected with expression constructs of LacZ or Siah2 myc into HEK293T cells. Lysates were collected after 24 h post transfection and processed for Western blot analysis. Quantification from *n* = 3 independent experiments shows fold change in mean intensity measurements (Li-COR Odyssey CLx) compared to LacZ control. *P* value derived from an unpaired one-tail student’s *t* test. **e** Hedgehog response luciferase assay assessing the role of Siah2 in regulating Shh signaling output. Isolated P6 CGNs were nucleofected with the indicated constructs and cultured on matrigel coated white optical bottom plates for 48 h. Luciferase detection was performed using the Pierce Luciferase Glow kit and measured on the Pherastar FSX plate reader. Quantification from *n* = 4 independent experiments shows the fold change of the luciferase RLU relative to non-stimulated cells. *P* value derived from an unpaired one-tail student’s *t* test. Scale bar = 10 μm. Error bars represents mean ± SD.

To dynamically confirm whether primary ciliogenesis in response to Shh required Ras or Siah2, we developed a confocal time-lapse microscopy assay to visualize this process (Fig. 5a and Supplementary Videos 2–5). In this assay, we nucleofected GNPs isolated from P7 WT cerebella with constructs encoding venus tagged Arl13b, the FUCCI fragment of human geminin^47^, an S-G2-M cell-cycle marker, and nuclear BFP–tagged shRNA. Cells expressing all three constructs were selected at the beginning of the time-lapse study and tracked for 14 h, during which time we could monitor the cells as they underwent cell-cycle progression, mitosis, and primary ciliogenesis (Fig. 5b). In control GNPs, in the absence of Shh-N, a high proportion of the cells failed to undergo primary ciliogenesis after cell division, whereas ciliogenesis was greatly increased in the presence of Shh-N, showing that Shh signaling promotes GNP primary ciliogenesis. When Ras or Siah2 were silenced in the presence of Shh-N, the proportion of cells that failed to undergo primary ciliogenesis increased relative to that in control Shh-N-treated cells, showing that Shh signaling-driven primary ciliogenesis requires Ras or Siah2.

We next focused on understanding how Siah2 controlled ciliogenesis in GNPs. Siah2 recognizes a well-defined degron motif^48^ in its targets; thus, we reasoned that Siah2 could potentially promote primary ciliogenesis by targeting proteins that promote cilium disassembly for degradation. Therefore, we scanned the literature for candidate proteins that played a role in cilium disassembly and contained Siah2 degron motifs, with a view to using them in a screen to complement ciliation phenotypes related to the activation of Shh signaling or Siah2 gain of function. We identified four candidates: the F-actin cross-linking protein α-actinin 4 (Actn4); the actin binding protein Dbn^49^; a microtubule-depolymerizing kinesin (Kif19a)^50^; and the protein encoded by the mouse embryonic node gene *Pitchfork* (Pifo), which exists in two isoforms: the full-length isoform (PifoL) and a shorter isoform (PifoS)^51^. We generated constructs encoding each Siah2 target protein and nucleofected GNPs isolated from P7 WT cerebella in conjunction with the bicistronic vector encoding a Venus-tagged Arl13b and mCherry-tagged histone H2B, then we grew the cells in culture in a serum-free condition. Also included in the screen was the polarity protein Pard3, a known target of Siah2 (Supplementary Fig. 8). The bicistronic construct enabled us to perform live-cell imaging and to determine the percentage of ciliated cells among the nucleofected cells. In this screen, we identified PifoS, Pard3, and Dbn as targets of Siah2 that could diminish Shh-driven primary ciliogenesis. To mitigate the possibility that overexpression of the Siah2 targets may titrate away the function of Siah2 through increased presence of Siah degron sequences alone we repeated this live-cell ciliogenesis assay using the PifoS, Pard3, or Dbn mutant forms where the Siah degrons have been mutated to abrogate Siah2 binding. We found that both WT and mutant forms of the Siah2 targets all inhibited ciliogenesis when coexpressed with Siah2 (Fig. 5c). To confirm that PifoS is a target of Siah2, we mutated the two Siah degrons present near the N-terminal of PifoS and co-expressed the WT or mutant version with Siah2 or its dominant negative version Siah2ΔRING in HEK293T cells. Mutation of the Siah degrons increased PifoS abundance suggesting the degron loss rendered it resistant to degradation by WT Siah2 (Fig. 5d). Moreover, expression of Siah2ΔRING increased the abundance of both PifoS wt and its degron mutant.

Thus far, our data showed that Siah2 regulates ciliogenesis which suggests its direct involvement in controlling Shh signaling. To confirm this, we performed a Hedgehog response luciferase assay to assess the effect of Siah2 overexpression or knockdown on Shh signaling. We generated a Shh response luciferase construct by subcloning the 8× Gli binding site multimer developed by Sasaki et al.^52^ into the pGL4.24 construct from Promega. We co-expressed this construct with shRNA against Siah2 or Siah2 expression construct in isolated CGNs from P6 cerebella and stimulated the cells with control or Shh-N conditioned media for 48 h (Fig. 5e). We found that Siah2 knockdown diminished, while Siah2 overexpression enhanced Shh responses. Taken together, these data show that primary ciliogenesis in response to Shh requires Ras or Siah2 and that Siah2 regulates primary ciliogenesis by blocking the function of Pard3, Dbn, and targeting the cilium disassembly protein Pifo for degradation.

### Primary ciliogenesis requires laminin and integrin β1-Ras

Having gathered evidence that the apparent regulation of Shh signaling by Ras–MAPK and Siah2 via ciliogenesis, we next sought to identify other extrinsic signals that could modulate this process via Ras–MAPK in the developing EGL GZ. Ras–MAPK is classically activated by growth factors such as EGFs or IGFs and their tyrosine kinase receptors^53,54^. In situ data from the Brain Transcriptome Database show that the pia surrounding the EGL of a P7 cerebellum expresses insulin-like growth factor 2 (Igf2) (Supplementary Fig. 9A). Given that Igf2 activates the Ras–MAPK cascade and that the high concentration of insulin in the neuronal culture supplement B27 triggers the Igf2 receptor, we wanted to rule out the possibility that Igf2 signaling controlled primary ciliogenesis and Siah2 expression. Isolated P7 CGNs from WT cerebella were placed in culture in insulin-free medium and stimulated with Igf2, Shh-N, or both in combination for 48 h (Supplementary Fig. 9B). The effect of Igf2 on ciliogenesis was negligible compared to that of Shh-N. These data are consistent with previous reports that other receptor tyrosine kinase receptor ligands have negligible mitogenic activity for GNPs when compared to Shh^10^.

**Fig. 6:**
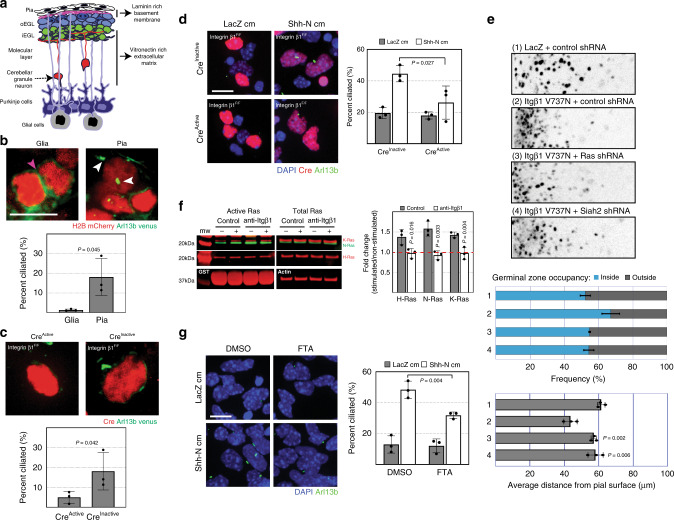
Laminin supports primary ciliogenesis through integrin β1-Ras. **a** Stratification of the early postnatal cerebellum. Distinct components make up the extracellular matrix of the EGL. The cerebellar pia mater secretes a laminin-rich extracellular matrix with which proliferating GNPs make contact, whereas deeper in the cerebellum, differentiated CGNs contact a vitronectin-rich extracellular matrix. The mitogenic effect of Shh is promoted by laminin but inhibited by vitronectin. Schematic generated by TO. **b** Isolated P7 CGNs were nucleofected with the bicistronic vector carrying Venus-tagged Arl13b and mCherry-tagged histone H2B and were plated on monolayers of age matched cerebellar glial or pial cells. After 24 h in culture, the cells were fixed and processed for immunofluorescence staining. Quantification (on Glia, *n* = 300; on pia, *n* = 525; individual cells from 3 independent experiments) shows the proportion of nucleofected cells that were ciliated. Magenta and white arrowheads denote the membrane and primary cilia, respectively. **c**, **d** P7 CGNs isolated from *integrin β1*^*F/F*^ cerebella were co-nucleofected with constructs expressing Venus-tagged Arl13b and a bicistronic vector carrying either the active or inactive mutant of Cre recombinase and mCherry-tagged histone H2B. The cells were cultured for 24 h on monolayers of age-matched pial cells (**c**) or laminin-coated slides with or without Shh-N as indicated, cm = conditioned medium (**d**). After 24 h in culture, the cells were fixed and processed for immunofluorescence staining. Quantification of *n* > 300 independent cells from 3 independent experiments shows proportion of nucleofected cells (H2B mCherry+) that were ciliated. **e** Ex vivo cerebellar pulse-chase assay assessing the effects of integrin β1 sensitization on GZ exit. Quantifications of *n* > 7000 independent cells in each condition from 3 independent experiments show the mean frequency distribution within and outside the EGL (top panel) and the average migration distance (lower panel). Horizontal length of representative images represents radial distance of 300 μm from cerebella pial. **f** Isolated P6 CGNs were plated on laminin coated plates and pre-treated with or without integrin β1 blocking antibody. Cells were stimulated with Shh-N or control conditioned medium for 15 min followed by the collection of whole cell lysate which were subjected to active Ras pull-down and immunoblotting for Ras isoforms. Quantification from three independent experiments shows the active Ras mean fold change relative to control stimulated cells. Full scans of blots available in Supplementary Fig. 11C. **g** Isolated P7 CGNs were plated on laminin coated glass and cultured as indicated, for 48 h prior to fixation and immunofluorescence staining. Quantification of *n* > 1000 independent cell in each condition from 3 independent experiments shows mean percent primary ciliated cells. *P* values derived from an unpaired one-tail student’s *t* test comparing—(**b-c**) groups; **e** experimental groups vs. Group 2; **f** control vs. anti-Itgβ1; or as indicated. Scale bar = 10 μm. Error bars represents mean ± SD.

GNPs express integrin β1 and interact with the laminin-rich basement membrane secreted by the pia surrounding the developing cerebellum^3,5^ (Fig. 6a). Interestingly, GNPs lacking integrin β1 are insensitive to Shh^3^ suggesting a critical role of matrix interactions in maintaining GNP responsiveness to a critical mitogen. We proceeded to place isolated CGNs from P7 cerebella in culture on monolayers of dissociated pial epithelia or glia from age-matched cerebella (Fig. 6b). After 48 h in culture, by which time most cells lose their primary cilia, a greater proportion of the GNPs on the pial monolayers were ciliated when compared to those on the glial monolayers. Next, we investigated whether primary ciliogenesis in GNPs on pial monolayers was dependent on integrin β1. Isolated CGNs from P7 *Integrin β1*^Flox/Flox^ cerebella were nucleofected with constructs encoding Cre recombinase or an inactive Cre mutant as a control, together with a Venus-tagged Arl13b to mark the primary cilia. The nucleofected cells were then placed in culture on pial monolayers for 48 h (Fig. 6c). The results showed that when integrin β1 was deleted from GNPs, fewer primary cilia were detected.

Having determined that the pia supported primary ciliogenesis in GNPs, we wished to determine whether this was mediated by laminin, the ECM substrate secreted by the pia. Conditional deletion of laminin from the pial basement membrane decreased proliferation in GNPs^5^, suggesting that there was a defect in primary ciliogenesis. Accordingly, we plated isolated CGNs from P7 WT cerebella on laminin- or vitronectin-coated glass in the presence or absence of Shh-N. The latter substrate is found in the ECM surrounding the iEGL and the ML (Supplementary Fig. 10A), where differentiated CGNs reside and has an inhibitory effect on Shh^6^. We found that laminin, but not vitronectin, supported the effect of Shh-N on both Siah2 expression (Supplementary Fig. 10C) and the primary cilia (Supplementary Fig. 10D) in GNPs. To determine whether integrin β1 was required for primary ciliogenesis when GNPs were plated on laminin, we nucleofected constructs encoding Cre recombinase, or an inactive Cre mutant as a control, into isolated CGNs from P7 *Integrin β1*^Flox/Flox^ cerebella and plated them on laminin in the presence or absence of Shh-N. When integrin β1 was deleted, fewer primary cilia were detected in response to Shh-N (Fig. 6d), and Siah2 expression was decreased (Supplementary Fig. 11A).

Given that integrin β1 signaling is required for primary ciliogenesis, we wanted to know whether enhanced integrin β1 signaling would affect GZ exit in developing GNPs. To test this, we electroporated the EGLs of P7 WT cerebella with constructs encoding the auto-clustering mutant integrin β1 (V737N)^55^ or LacZ as a control. We found that enhanced integrin β1 signaling blocked GZ exit. We also knocked down Ras or Siah2 under the condition of enhanced integrin β1 signaling and found that GZ exit was rescued, showing that Ras or Siah2 functions downstream of integrin β1 in controlling GZ exit (Fig. 6e). We note that in the in vitro transfection conditions tested expression of the RasV12T35S (Ras–Raf), a Raf–Mapk activating Ras mutant but not integrin β1 V737N was sufficient in the absence of Shh signaling to maintain Siah2 expression (Supplementary Fig. 10B), albeit at levels below which we observed for Gli1 or SmoM2 expression (Fig. 2).

We show that CGNs plated on laminin and stimulated with Shh-N conditioned medium activated Ras (Supplementary Fig. 4) suggesting that Shh and integrin β1 may signal in a similar pathway to activate Ras. To test this, we pre-treated isolated CGNs from P7 cerebella plated on laminin, with or without integrin β1 blocking antibody. The cells were stimulated with Shh-N conditioned medium for 15 min and lysates were subjected to active Ras pull-down followed by immunoblotting of Ras isoforms (Fig. 6f). We found that blocking integrin β1 abrogated Shh-N activation of Ras. In addition, the Ras inhibitor FTA when added to P7 GNPs stimulated with Shh-N resulted in fewer primary cilia (Fig. 6g) and decreased Siah2 expression (Supplementary Fig. 11B). Finally, to further assess that both Shh and Integrin β1 function in a similar pathway we performed epistasis experiments where we activated Shh, integrin β1, or Ras–Raf pathways singly or in combination to assess if there were additive effects on GZ exit (Supplementary Fig. 12). We found that combined activation of the Shh and integrin β1 or Shh and Ras–Raf did not further impair GZ exit when compared to Shh, integrin β1, or Ras–Raf activation singly, confirming that these components function in a similar pathway. Taken ether, these data show that the pial epithelium and the laminin that it produces provide a supportive microenvironment that promotes primary ciliogenesis in GNPs, enhancing their response to Shh.

## Discussion

Throughout the developing nervous system, GZ occupancy by proliferating progenitor cells and the migration of postmitotic neurons are viewed as transient and independent phases of neuronal maturation, but there is limited insight into how these developmental stages are interconnected or potentially cross-regulated. For example, although it is apparent that radial migration is a process concomitantly linked with the cessation of progenitor proliferation and the start of neuronal terminal differentiation, it remains unknown how the cell biological programs that are necessary for migration initiation are both restrained in progenitor cells and stimulated by differentiation. It is also unclear how the cell-intrinsic machinery that organizes these processes is in turn regulated by cell-extrinsic signals, such as the mitogens that promote continued proliferation, or the contextual niche components, such as the ECM. The Siah2 E3 ubiquitin ligase appears to act as a central regulator of GZ occupancy by modulating two distinct cell biological processes. Previous results have shown that Siah2 restrains radial migration in progenitors by tagging for ubiquitin-proteasome degradation targets that are required for the pro-migratory adhesive interactions via Pard3 and actin–microtubule crosslinking via Dbn that are essential for GZ exit and migration along glial fibers^16,20^. The current study has extended these findings to implicate Siah2 as an active participant in GZ occupancy by supporting primary cilium maintenance in GNPs: Siah2 targets Pard3, Dbn, and Pifo for degradation, thereby ensuring the reception of signals from the Shh morphogen that promote GNP proliferation at the expense of CGN differentiation. Furthermore, the engagement of the laminin ECM substrate by integrin receptors supports Ras–Mapk activity in concert with Shh that is necessary for primary ciliogenesis, illustrating how coincidence detection between niche components converges on Siah2 to link GZ occupancy with proliferative signals. Intriguingly, Siah2 antagonism of Pard3 represents a mechanistic convergence point of GZ niche and mitogen coincidence detection. As Pard3 counters ciliogenesis and promotes CGN GZ exit, the relief of Siah2-target inhibition in differentiation represents a direct integration of responsiveness to a mitogen and migration initiation as GNPs mature into CGNs.

In this study, we have revealed an unexpected connection between Shh signaling, which maintains GNPs in the undifferentiated state, with Ras–Mapk and Siah2. Not only do GNPs display elevated phospho-Mek1/2 staining in the EGL in vivo, but FACs isolated GNPs purified from the EGL niche also have elevated levels of N-Ras activity and elevated N-Ras expression. While the exogenous application of Shh to acutely isolated EGL cells in vitro leads to an increase of N-Ras activity, suggesting Shh contributes to the elevated levels of Ras activity in GNPs, the short 15-min exposure to Shh does not stimulate elevated levels of N-Ras protein expression. Siah2 expression is not only maintained by Shh in a Ras–Mapk-dependent manner but is part of a functional feed-forward loop as Siah2 is in turn required for GNPs to sense a Shh signal. Although our study has not established Siah2 as a direct target of the Shh pathway, there are clear canonical aspects to Shh maintenance of Siah2 expression: (1) the gain of function associated with Shh activators such as SmoM2 and Gli1 shows that the Shh pathways are sufficient to maintain Siah2 expression; and (2) Shh-induced maintenance of Siah2 requires the primary cilia and Gli transcription factors. It is important to note that genome-wide ChIP analyses in developing GNPs have identified Siah2 as a binding target of Gli1, supporting our findings that Gli1 activates Siah2 expression^56^ and that Gli transcription factors are necessary for Shh-N stimulation of Siah2 expression. Shh maintenance of Siah2 expression differs from primary Shh targets such as Ptch and Gli1 that are activated almost immediately after Shh addition^57,58^, because the effects of Shh on Siah2 expression required 36–48 h of exposure. This unique temporal variation on the usual theme of Shh-induced changes in gene expression may stem from the fact that GNPs isolated during the peak proliferative period have been pre-exposed to Shh in vivo and, hence, exhibit peak Siah2 expression. It will be interesting to determine if long-term Shh exposure stimulates N-Ras expression in GNPs, as observed for other cell cycle regulatory proteins like D-type cyclins^59^ or the Myc proto-oncogenes^57,60^, and if cilia and Gli transcription factors are involved similar to Siah2.

Our study has broadened our knowledge of the previously reported crosstalk between Ras/Mapk and Shh signaling. Oncogenic Ras/Mapk have been demonstrated to promote the stability and transcriptional activity of Gli transcription factors in pancreatic ductal adenocarcinoma (PDAC)^61^. Moreover, Ras/Mapk activity is required for Gli protein stability and nuclear translocation in melanomas^62^. In both of these cases, the loss of Ras/Mapk- or Gli1-mediated Shh signaling resulted in decreased cell proliferation similar to that which we observed in GNPs, suggesting that Shh and Ras/Mapk signaling must co-exist for optimal proliferative output. The precise mechanistic pathways regulating this crosstalk are poorly characterized in PDAC and melanoma. Our finding that Ras/Mapk is required for ciliogenesis suggests that maintenance of this critical Shh-transducing organelle may underlie some of the Ras/Mapk support of Shh signaling, although it is important to note that PDACs are devoid of primary cilia^63^.

The process of primary ciliogenesis involves a dynamic orchestration of events, and the molecular mechanisms by which this process is regulated have been delineated using culture systems of cells that primarily elaborate cilia in a quiescent or postmitotic state^64^. In these models, it is thought that the primary cilium impedes cell-cycle progression and that its loss is required for re-entry into the cell cycle. A major concern with these studies, which often use serum starvation to induce primary ciliogenesis, is their lack of physiologic relevance or applicability to the mechanisms of ciliogenesis in vivo. Contrary to fibroblast cells, which are commonly used to study primary ciliogenesis where primary cilia retraction is associated with cell-cycle re-entry, neural stem cells can shed their primary cilia through apical abscission to enter quiescence^65^, while some rapidly dividing tumor cells are known to possess primary cilia when in the actively cycling state^66,67^. Our data support the shift in dogma by revealing that the primary cilia persist well into the S, G2, and early M phases of the GNP cell cycle, a transiently amplifying progenitor cell similar to basal progenitors in the cerebral cortex. The structure is quickly disassembled for a short time during late M phase as a result of the process of cytokinesis, but primary cilia reappear rapidly in GNPs as they continue to proliferate if Shh is present. This suggests that the primary cilia in GNPs facilitate cell-cycle progression and, given the highly proliferative nature of these cells, that it is crucial for them to maintain their primary cilia to continuously sense Shh. This is best substantiated by the findings that MBs that rely on high Shh signaling requires the primary cilia^66^. We can extend this concept beyond GNP proliferation to encompass CGN migration initiation, as sustained Shh signaling and cilia shortening have opposing effects on GZ exit. Thus, not only do GNPs maintain their primary cilia in order to remain sensitive to Shh, as we have shown, but differentiated CGNs shorten their primary cilia, rendering the cells insensitive to Shh and enabling them to undergo radial migration.

Mechanistically, our work has revealed a feed-forward mechanism by which Shh signaling maintains primary ciliogenesis in proliferative GNPs. Elevated Siah2 expression in the presence of Shh supports primary ciliogenesis after GNP cell division as Siah2 is both necessary for ciliogenesis in Shh-treated GNPs and, when over-expressed, sufficient by itself to maintain cilia. How does Siah2 contribute to ciliogenesis? The substrate-binding domain of Siah ligases forms a binding groove that specifically recognizes its substrates through a degron motif: Px[ARTE]xVxP^48,68^ that has remained mostly unchanged from invertebrates to humans. The high level of Siah2 expression in GNPs suggests a model whereby a group of Siah degron-containing target proteins are degraded in progenitors that promote cilium retraction in CGNs when Siah activity diminishes during differentiation. Our small-scale Siah2 target-expression search for proteins that could promote diminished primary cilia revealed that Pard3, Dbn, and Pifo expression in Siah2-overexpressing CGNs led to cilium disassembly. In contrast, the Siah degron containing alpha-actinin 4, a target that is involved in uncontrolled ciliary membrane shedding in ciliopathies^49^, did not induce cilium disassembly in GNPs. We further established that the ciliary disassembly function of Pifo was limited to its shorter isoform, which suggests that the contradictory reports on the functions of Pifo^51,69^ may be attributed to its isoforms. The effect of Pard3 on neuronal progenitor cilia was surprising because this protein promoted cilium assembly in cells in culture that display ciliogenesis in the quiescent state^70^, suggesting that the mechanisms of ciliogenesis in cells that maintain their cilia while actively cycling are different from those of the classical cell types that grow cilia when quiescent. Moreover, the Pard complex-dependent cilium-disassembly activity and its regulation by Siah2 that was revealed by our study represents a rapid post-translational mechanism regulating GZ occupancy, whereby cilium status, polarization during differentiation, and the adhesive mechanisms are tuned via Siah2–Pard3 antagonism. Interestingly, both Pifo and Pard3, in combination with its binding partner aPKC, regulate the levels of Aurora A kinase^51,71^, which is a known regulator of cilium disassembly during the cell cycle, suggesting that both Pifo and Pard3 may impinge on known pathways for cilium disassembly via Aurora A^72^.

Previous studies demonstrated that the EGL serves as a mitogenic niche for developing GNPs and that migration away from this lamina is necessary to drive cell-cycle exit and cellular differentiation^73^, hinting that the microenvironment plays a key role in controlling these transformations; however, the mechanism remains elusive. In this study, we have demonstrated that laminin, a main component of the ECM surrounding the developing EGL, supports Shh signaling in GNPs^5,6^, and we have further determined that laminin cooperates with Shh signaling to maintain primary ciliogenesis in GNPs. Furthermore, Siah2 is a central regulator of ECM-Shh crosstalk by virtue of being downstream of Shh and Ras–Mapk, respectively, which ultimately regulates the window of GNP responsiveness to a mitogen via an unexpected function in ciliogenesis. Interestingly, laminin is present only in the basement membrane surrounding the EGL that proliferating GNPs contact^5,6^. The laminin-rich ECM transitions to vitronectin deeper in the iEGL and ML^6,74^. We have provided data showing that vitronectin counters the mitogenic effect of Shh by blocking primary ciliogenesis in GNPs demonstrating an exquisite specificity of ECM to Shh coincidence detection: while both integrin β1 involved in laminin^75^ binding and integrin β3 involved in vitronectin^76^ binding both signal through the Ras–MAPK cascade only laminin supports Shh-induced ciliogenesis. Thus, GNPs possess an ECM–mitogen coincidence detection machinery upstream of Siah2 that is sufficient to discriminate between varied Ras–Mapk activators. Such ECM–Shh coincidence detection is likely to reside outside of the nervous system as primary ciliogenesis in the developing dermal papillae epithelial cells requires integrin β1 and its interaction with laminin but is inhibited by integrin β3^77^. Given Siah2 recently expanded role in controlling epithelial polarization^78^ it will be exciting to determine if ECM regulation of Siah2 activity plays a similar role in developing epithelia.

Given the pattern of migration in developing CGNs, the transition in components of the ECM represents a unique mechanism for controlling the fine transition between cellular proliferation and differentiation. Further studies are required to determine whether there are cell-autonomous factors that can override the cooperative effects of laminin and Shh. One interesting observation stems from the fact that most Ptch-deficient GNPs eventually exit the EGL and undergo differentiation^79^. One could postulate the existence of a molecular timer in GNPs that limits their proliferative capacity and must be overcome in the event of tumorigenesis.

The newly discovered crosstalk between Shh, ECM, and Siah2 may be relevant to GZ-exit defects in cerebellar tumorigenesis. Shh–MBs arise from GNPs with aberrant Shh signaling^8–10^. In humans, these tumors also exhibit increased Siah2 expression, as compared to that in other subgroups and they are ciliated. Various mouse models, and specifically the Ptch-deficient mouse model^43^, have faithfully recapitulated this subgroup of tumors. By using a similar mouse model, we have shown that acute deletion of *Ptch1* in GNPs blocked GZ exit, thereby recapitulating the overt phenotype seen in these preneoplastic cells. More importantly, we have shown that this phenotype is caused by the increased expression of Siah2 and that it was rescued when Siah2 was knocked down or when its function was inhibited. Overall, our data delineate a molecular mechanism that explains why GZ exit is affected in preneoplastic GNPs and why these tumor subtypes maintain their primary cilia. Given the ongoing efforts to develop Siah inhibitors^80^, the results of this study make Siah2 an interesting potential target for treating human Shh–MB, given that reducing Siah2 activity accelerates differentiation and blocks Shh-driven primary ciliogenesis; thus, Siah inhibition has potential for use in differentiation-inducing therapies for MB.

## Methods

### Ex vivo cerebellar pulse-chase assay

P7 cerebellar were dissected, soaked in a suspension of plasmid DNA in Hank’s buffered salt solution (HBSS) at a concentration range of 1–3 μg/μL per construct (the nuclear marker H2B-mCherry was always included to track cellular migration), and electroporated in a platinum-block petri-dish electrode (CUY520-P5; Protech International) by using a square-wave electroporator (CUY21-EDIT; Protech International) with the following program: 5 pulses, 90 V, 50-ms pulse, 500-ms interval. The electroporated cerebella were embedded in 4% low-melting-point agarose in HBSS, and 300-μm sagittal sections of the cerebellar vermis were prepared on a vibratome (VT1200; Leica microsystems). The sections were transferred to 0.4-μm Millicell cell culture inserts (Millipore) and incubated in Basal Medium Eagle supplemented with 0.5% glucose, 2 mM l-glutamine, 50 U/mL penicillin–streptomycin, and 1× B27 and 1× N2 supplements (ThermoFisher). After 24 or 48 h, the slices were fixed with 4% paraformaldehyde then stained with EdU, if necessary, and mounted on glass slides, with ProLong Gold antifade mountant (ThermoFisher) being applied before image acquisition. To measure the migration distance, the coordinates of the center of each nucleus marked by H2B-mCherry were first determined using SlideBook 6.0.15 imaging software (Intelligent Imaging Innovations). The coordinates were then exported into the IGOR Pro 7.0.8 line analysis software (WaveMetrics Inc.), which measures the perpendicular distance of each nucleus from the surface of the cerebellum. Statistical analyses were performed using Excel.

### Isolation and nucleofection of CGNs

P7 cerebella were dissected and dispersed using the Neural Tissue Dissociation Kit P (Miltenyi) as recommended. The tissue was titurated into a single-cell suspension by using fire-polished glass Pasteur pipettes. CGNs were isolated by centrifugation over a discontinuous Percoll gradient, and the higher-density fraction, which contained 95% CGNs and 5% glia, was collected. CGNs were nucleofected with plasmid DNA by using the optimized Amaxa Mouse Neuron Nucleofector Kit (Lonza) with the O-005 program. The cells were recovered for 5 min then plated over poly-L-ornithine-, Matrigel-, laminin-, or vitronectin-coated 16-well slides or glass-bottom dishes (EMS) in Neurobasal medium supplemented with 0.5% glucose, 0.4 mg/mL of tissue culture-grade bovine serum albumin (Sigma), 2 mM l-glutamine, 50 U/mL penicillin–streptomycin, and 1× B27 supplement (ThermoFisher). When specified, insulin-free B27 supplement (ThermoFisher) was used in place of the standard B27 supplement.

### Image acquisition

Imaging was performed on a 3i Marianas Spinning Disk confocal microscope (Intelligent Imaging Innovations) consisting of a Zeiss Axio Observer microscope equipped with a 40/1.0 numerical aperture (NA) Plan-Apochromat (oil immersion) objective and a 63/1.4 NA Plan-Apochromat (oil immersion) objective. An Ultraview CSUX1 confocal head with 440–514 nm or 488–561 nm excitation filters and an ImageEM-intensified CCD camera (Hamamatsu) were used for high-resolution imaging. Images and video recordings were captured using SlideBook software (Intelligent Imaging innovations).

### Image segmentation, quantification, and statistical analysis

Analyses of confocal images were performed using Slidebook 6.0.15 (3i-Inteligent Imaging Innovations). 3D-EM analyses—image stacks were aligned, filtered, cropped, and resampled to 8 bit by using the FEI Amira 6.2.0 software package. Individual primary cilium was segmented manually. Quantified data are expressed as mean ± SD. Student’s *t* test was performed when comparing two groups with a statistical significant cutoff value of *P* = 0.05. The *χ*^2^-test with the statistical significant cutoff value of *P* = 0.05 was used to compare Ras or Siah2 knockdown effect on primary ciliogenesis compared to control in Fig. 5a.

### Fixation and processing for 3D electron microscopy

Sagittal sections of the cerebellum (300 μm in thickness) were fixed in 2% paraformaldehyde and 2.5% glutaraldehyde in 0.1 M cacodylate buffer overnight then rinsed in the same buffer. The tissue was stained using a modified heavy metal staining method then processed through a graded alcohol/propylene oxide series and infiltrated in propylene oxide/epon gradients. The tissue was then infiltrated overnight with 100% Epon 812 resin and polymerized for 48 h in an oven at 70 °C.

### Preparation steps for focused ion beam scanning scanning electron microscopy (FIB-SEM) imaging

The sample block was mounted on an aluminum pin stub with a conductive silver paint and sputter-coated with a thin (<60 nm) layer of iridium by using a Denton DeskV sputter coater in order to electrically ground the sample and limit charging. To locate the region of interest (ROI), the block face was scanned at an acceleration voltage of 10 kV and a current of 0.2 nA on a scanning electron microscope using a concentric backscatter detector. The block was trimmed to the ROI with a Leica UC7 ultra-microtome fitted with a DiATOME knife. A relief was then milled into the block face by using a suitable ion beam current, and a protective cap was deposited using the carbon gas injection system. Fiducials were created to aid the computer vision pattern-placement algorithm. Regions of interest were imaged using the FEI Auto Slice and View software package to automate the serial-sectioning and data-collection processes.

### Focused ion beam scanning electron microscopy

The samples were imaged and processed for 3D data collection on an FEI Helios G3 system. The imaging parameters were 2 kV, 0.4 nA, and 5 × 5 × 5 nm with an in-column detector.

### Western blotting

Cells were lysed in N-PER lysis buffer (ThermoFisher) supplemented with 1× Halt Protease and Phosphatase Inhibitor Cocktail (ThermoFisher). Lysates were reduced in Bolt Sample Reducing Agent (ThermoFisher) and LDS loading buffer (ThermoFisher) at 75 °C for 5 min. The samples were electrophoresed on a 4–12% sodium dodecyl sulfate (SDS) polyacrylamide gel electrophoresis gel (ThermoFisher) and electroblotted onto a 0.45-μm Immobilon-FL polyvinylidene fluoride membrane (Millipore) by using the Criterion Blotter (Bio-Rad). The membranes were blocked with a 1:2 dilution of Odyssey Blocking Buffer (LI-COR) in phosphate-buffered saline (PBS) for 1 h at room temperature then incubated with primary antibodies overnight at 4 °C. Odyssey IRDye secondary antibodies (LI-COR) were used for the detection on an Odyssey CLx scanner (LI-COR).

### Active Ras pull-down

Purified GNPs were pre-plated for 1 h to remove any adherent cells. Cells were collected by centrifugation at 100 × *g* for 5 min and rinsed once with ice-cold calcium- and magnesium-free PBS. They were then lysed in 1× lysis/binding/wash buffer supplemented with 1× Halt Phosphatase and Protease Inhibitor Cocktail (Thermo Fisher). Cell lysates were collected, and the protein concentration was determined using the Pierce BCA Protein Assay Kit (Thermo Scientific) and adjusted to 1 mg/mL. A total of 500 µg of total protein for each condition was treated with GTPγS or GDP at a final concentration of 10 mM for 15 min at 30 °C with constant agitation. The reaction was stopped by adding MgCl_2_ to a final concentration of 60 mM on ice, and the reaction mixture was transferred to a spin cup with glutathione resin and 80 μg of GST–Raf1–RBD, prepared according to the manufacturer’s instructions. The reaction mixture was incubated at 4 °C for 1 h with gentle rocking then washed three times with 1× lysis/binding/wash buffer. Reducing sample buffer was prepared by adding dithiothreitol to 2× SDS sample buffer to a final concentration of 200 mM. Samples were eluted with 50 µL of reducing sample buffer and heated at 95 °C for 5 min.

### Immunofluorescent staining

In vitro CGN cultures were fixed with 4% paraformaldehyde for 15 min and permeabilized with 0.2% triton x-100 for 10 min. Frozen tissue sections were washed briefly with PBS followed by permeabilization with 1% SDS for 20 min. This was followed by PBS washes and blocking for 30 min with 5% donkey serum. Slides were incubated overnight at 4 °C with primary antibodies diluted in PBS with 0.5% donkey serum and 0.02% Triton X-100. Alexa Fluor-conjugated secondary antibodies (ThermoFisher) were used to detect the primary antibodies and Prolong Gold Antifade Mountant (ThermoFisher) was applied to the slides before imaging.

### Tissue preparation for cryosectioning

Whole brains were dissected and fixed in 3% paraformaldehyde in PBS overnight at 4 °C with gentle shaking. Specimens were then cryoprotected with 30% sucrose in PBS before being embedded in NEG-50 medium (ThermoFisher). Sections (14 μm in thickness) were cut on a cryostat and collected on ColorFrost Plus microscope slides (ThermoFisher). Sections were dried on a slide warmer for 30 min prior to storage at −20 °C.

### Antibodies

The following antibodies were used at the dilutions specified—immunofluorescent (IF) or Western blot analysis (WB): anti-Arl13b, 1:2000 IF (clone N295B/66, UC Davis/NIH NeuroMab Facility), anti-Ac3 (gift from Young-Goo Han laboratory), anti-Siah2, 1:200 IF (Santa Cruz Biotechnology, cat. no. SC81787); anti-Tag1, 1:50 IF (UC Davis/NIH NeuroMab Facility, cat. no. 4D7/TAG1); anti-β-actin, 1:20000 WB (Sigma, cat. no. A2228); and anti-phospho-Mek1/2, 1:500 IF (Cell Signaling, cat. no. 9121); anti-H-Ras, 1:500 WB (Abcam, cat. no. ab32417); anti-N-Ras, 1:500 WB (Abcam, cat. no. ab77392); anti-K-Ras, 1:2000 WB (Proteintech, cat. no. 12063-1-AP); anti-vitronectin, 1:200 IF (Thermofisher cat. no. MA5-24083); anti-integrin β1 (Biolegend. Clone HMβ1-1); anti-myc 1:2000 WB (Thermofisher, cat. no. PA1-981); anti-HA 1:2000 WB (Biolegend, clone 16B12).

### Plasmid vectors

All cDNA encoding proteins of interest were subcloned into pCIG2. All shRNA used is miR-30 based.

### Animals

Wildtype C57BL/6J, Ptch1^Flox/Flox^ (B6N.129-*Ptch1*^*tm1Hahn*^/J), Integrin β1^Flox/Flox^ mice (B6;129-*Itgb1*^*tm1Efu*^/J), Atoh1-eGFP (B6.129S-*Atoh1*^*tm4.1Hzo*^/J) were obtained from The Jackson Laboratory. All mouse lines were maintained in standard conditions in accordance with guidelines approved by the Institutional Animal Care and Use Committee at St. Jude Children’s Research Hospital (protocol No. 483).

### Atoh1-eGFP FACS

CGNs were isolated from homozygous Math1-eGFP (B6.129S-*Atoh1*^*tm4.1Hzo*^/J) pups between P6 and P8, following the dissociation methods described. Acquisition and sorting were performed on a BD Biosciences 4 laser Aria Fusion with a 100 μm nozzle, 21 psi. GFP was excited using a 488 nm laser with a 530/30 filter. Diva software. Sorted cells were subjected to active Ras pull-down.

### Reverse transcription polymerase chain reaction (RT-PCR)

Total RNA was isolated from cultured CGNs using the Ambion RNA Aqueous Kit (ThermoFisher) according to manufacturer’s recommendations, followed by two-step real-time RT-PCR analysis on the ABI PRISM 7900 detection system using random hexamers, TaqMan Reverse Transcription Reagents, and SYBR green PCR master mix (Applied Biosystems) according to Singh et al.^81^.

### Shh response luciferase assay

A luciferase-based Shh signaling response construct was generated by subcloning the 8× Gli binding site multimer from Sasaki et al.^52^ into pGL4.24 from Promega within the KpnI and BglII sites. The construct was delivered into isolated CGNs by nucleofection and plated on laminin coated optical flat-bottom white 96-well plates (Corning). The Pierce Firefly Luciferase Glow Assay kit (ThermoFisher) was used in accordance to manufacturer’s recommendation. Luminescence was measured on the PHERAstar FSX plate reader (BMGlabtech).

### Statistics and reproducibility

Sample sizes, statistical analyses and *P* values for every experiment are as stated in the figure legends. Raw data from independent experimental replicates are provided in the Source Data. Statistical analyses were performed using Excel or Prism8. Graphs were generated using Prism8 or KaleidaGraph. A *P* value of <0.05 was considered significant.

## Supplementary information

Supplementary Information

Supplementary Movie 1

Supplementary Movie 2

Supplementary Movie 3

Supplementary Movie 4

Supplementary Movie 5

## Data Availability

Source data for figures are provided with the paper. All other data are available from the authors upon reasonable request. Source data are provided with this paper.

## Source data

Source Data
