# Supplementary material for: Siah2 integrates mitogenic and extracellular matrix signals linking neuronal progenitor ciliogenesis with germinal zone occupancy
Source: Nat Commun. 2020 Oct 20;11:5312. doi: 10.1038/s41467-020-19063-7 (PMC7576183; doi:10.1038/s41467-020-19063-7)

Supplementary Information For:

Siah2 integrates mitogenic and extracellular matrix signals linking neuronal progenitor ciliogenesis with germinal zone occupancy.

Taren Ong[1], Niraj Trivedi[1], Randall Wakefield[2], Sharon Frase[1], and David J. Solecki[1*]

[1]Department of Developmental Neurobiology, St. Jude Children's Research Hospital, Memphis, TN 38105, USA

[2]Cell and Tissue Imaging Center-EM, St. Jude Children's Research Hospital, Memphis, TN 38105, USA

*Correspondence: david.solecki@stjude.org

Supplementary Figures

Supplementary figure 1. Schematic of how Siah2 is regulated in developing CGNs. (**A**) The laminin-rich basement membrane surrounding the oEGL in a developing cerebellum promotes Shh signaling driven primary ciliogenesis in GNPs via Integrin β1 – Ras/Mapk signaling. The primary cilium allows GNPs to sense the Shh mitogen and activate the Shh pathway to maintain Siah2 expression which in turn promotes GZ occupancy by inhibiting GZ exit. Siah2 acts in a feed-forward mechanism to maintain mitogen sensitivity by promoting primary ciliogenesis through the antagonism of a key cilia disassembly proteins Pifo and Dbn, and the polarity inducer Pard3. As GNPs leave the oEGL, the lack of trophic support leads to the disassembly of the primary cilium and loss of sensitivity to Shh, which promotes CGN differentiation. Schematic generated by TO. (**B**) Full scans of western blots from **Figure 1D.** Dotted-lined box shows the cropped locations.

Supplementary Figure 1

A

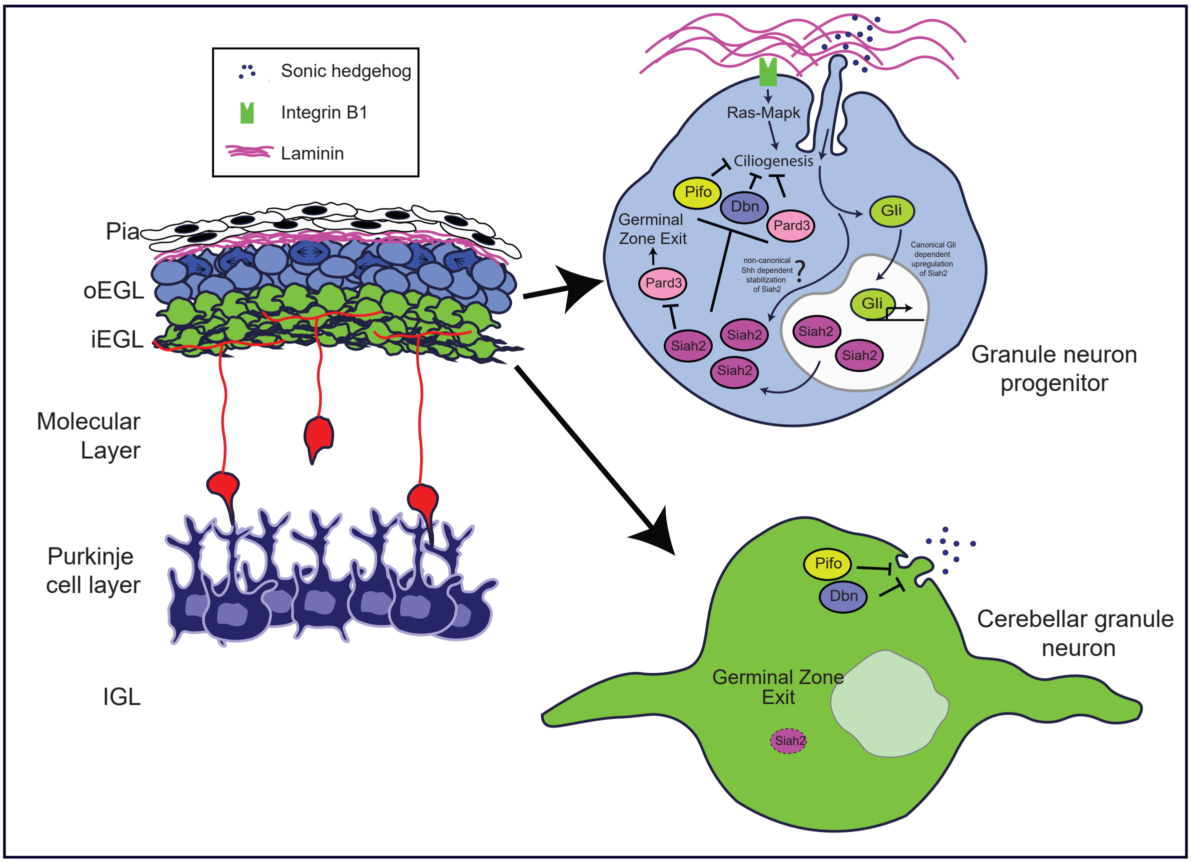

B

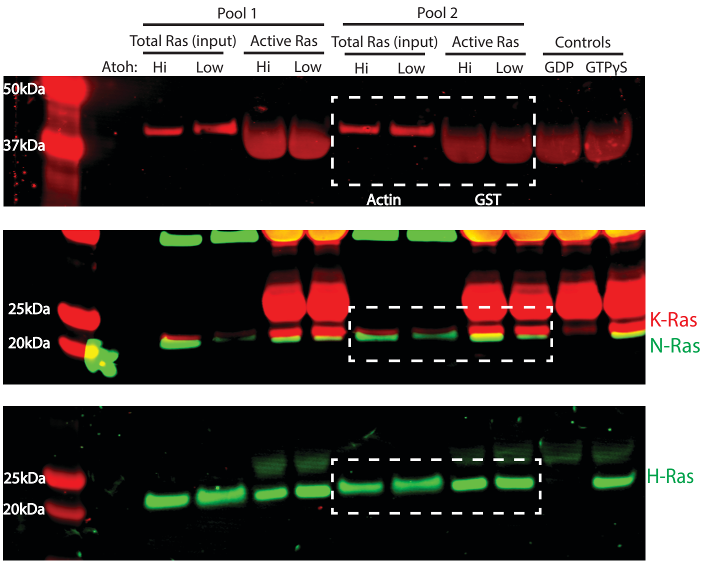

Supplementary figure 2. Canonical Shh signaling maintains Siah2 expression. Isolated P7 CGNs were: - (**A**) co-nucleofected with the nuclear marker H2B-mCherry and IFT88 shRNA and cultured *in vitro* on matrigel coated glass for 48 h in the presence or absence of recombinant Shh-N (3µg/mL) followed by immunofluorescent staining. Quantification n > 700 independent cells in each condition from 3 independent experiments shows mean percent ciliated cells based on Arl13b staining; (**B**) cultured on matrigel coated glass with or without Shh-N conditioned medium (cm) and supplementation of Gli inhibitors for 48 hours followed by fixation and immunofluorescence staining. Quantification n = 27 confocal image fields in each condition from 3 independent experiments shows mean percent Siah2 positive cells. (**C**) Isolated P7 CGNs were plated on poly-ornithine treated glass and stimulated with smoothened agonist (SAG 100nM) for 24-hours. mRNA was extracted for q-PCR analyses of Gli1 and Siah2 transcripts. Quantification from 3 independent experiments show relative Gli and Siah2 transcripts levels. *P* values derived from an unpaired one-tail student's t-test comparing – experimental groups (**C**) or to "Shh-N cm" (**B**); or as indicated. Scale bar = 10µm. Error bars represents mean ± SD.

Supplementary Figure 2

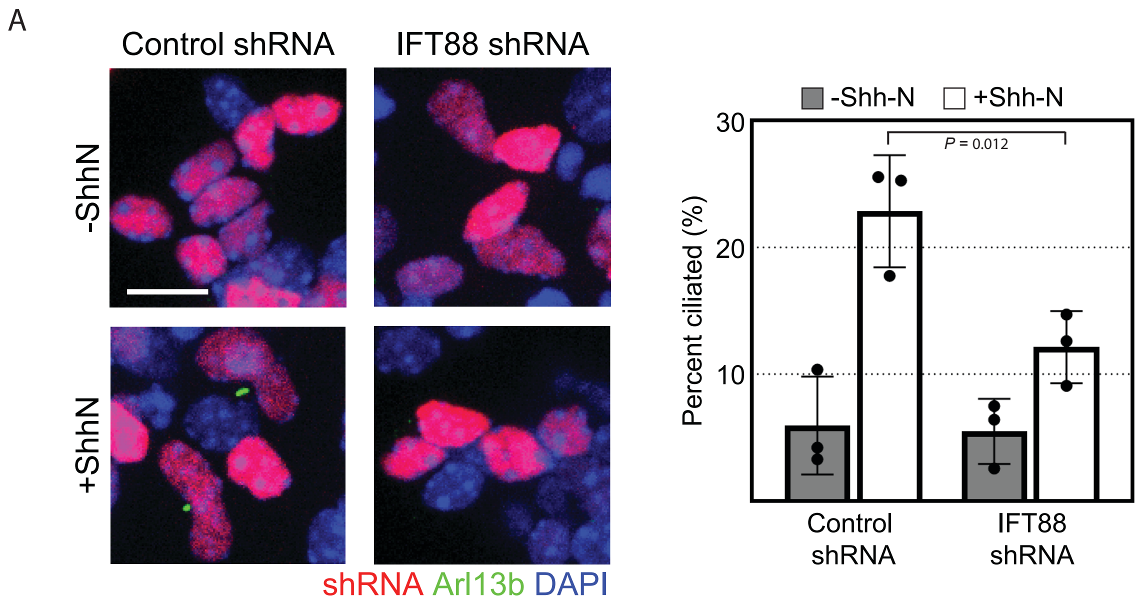

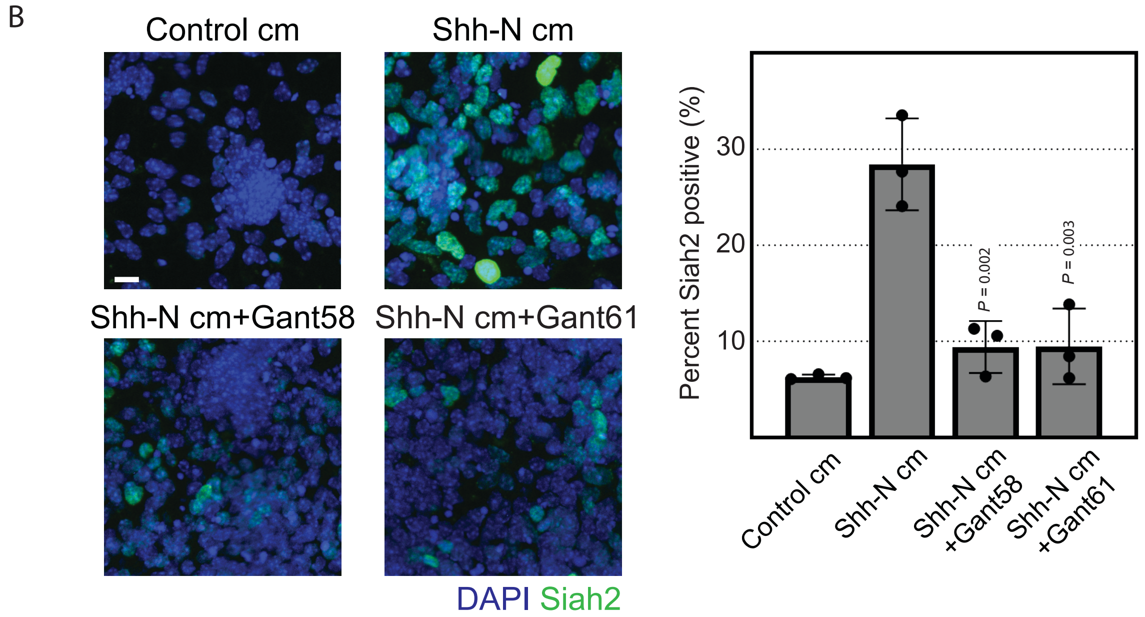

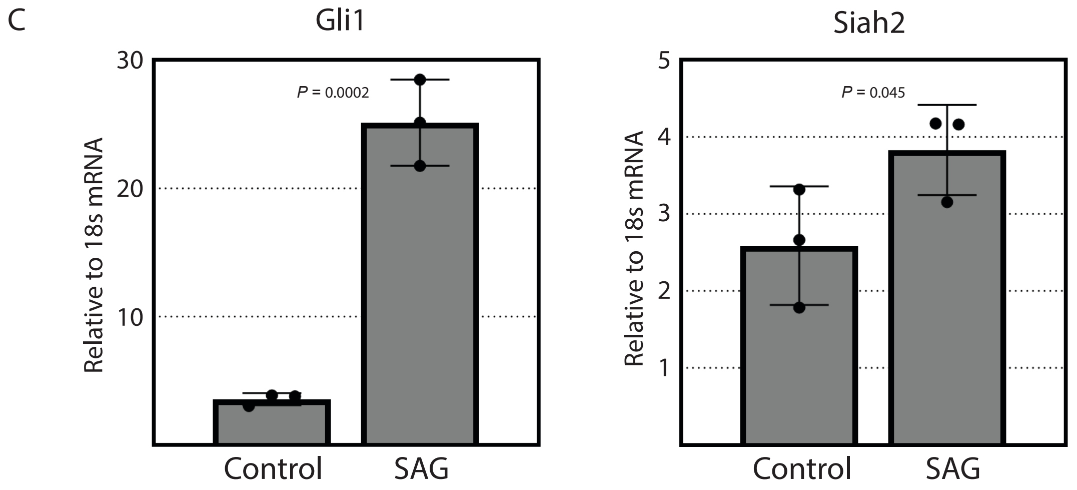

Supplementary figure 3. Shh maintenance of Siah2 requires the Ras-Mapk cascade. (A) Isolated P7 CGNs were plated on matrigel coated glass and cultured for 48 hours in the presence or absence of Smoothened agonist (SAG, 100nM) and with the indicated combinations of Ras/Mapk inhibitors: farnesyl transferase inhibitor (FTA), sorafenib (Sor), trametinib (Tram), and FR180204 (FR), which inhibit Ras, Raf, Mek1/2, and Erk1/2, respectively. The cells were fixed followed by immunofluorescence staining of Siah2. Quantification Control (n=18); all other conditions (n=24) independent confocal image fields from 4 independent experiments shows the mean percent Siah2 positive cells determined using SlideBook. *P* values derived from an unpaired one-tail student's t-test comparing experimental groups to "SAG". Scale bar = 10μm. Error bars represents mean ± SD. (**B**) shRNA validations (n = 3 independent experiments) were performed by co-transfecting HEK293T cells with plasmids encoding control or the indicated shRNAs and their corresponding target's expression constructs. Cell lysates were collected 24 hours post transfection for immunoblotting analyses.

Supplementary Figure 3

A

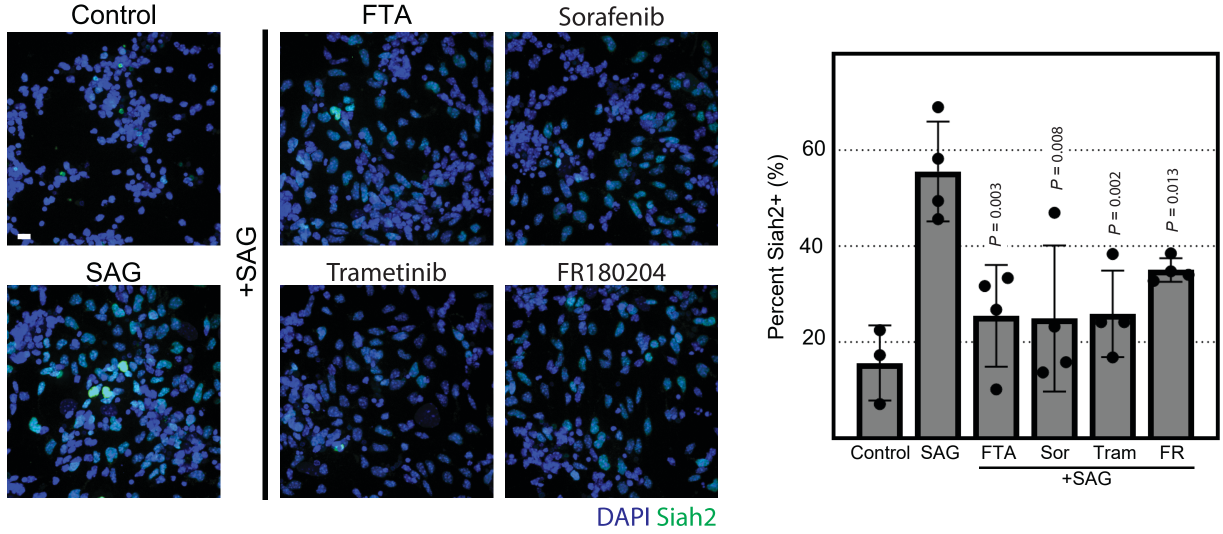

B shRNA validation

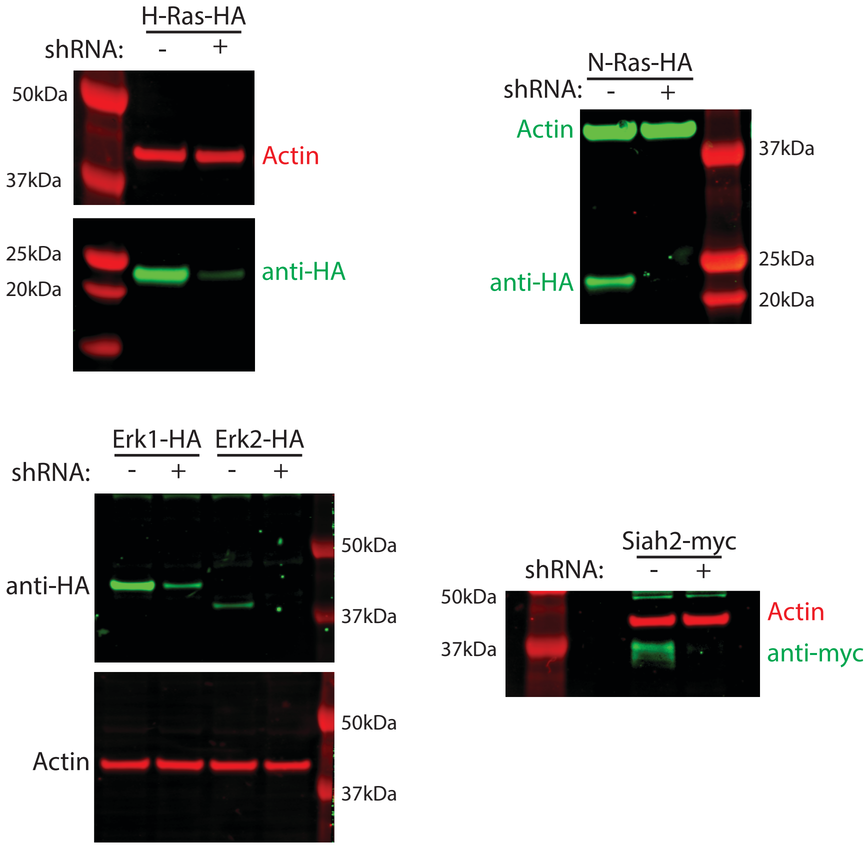

Supplementary figure 4. Shh stimulation raises the levels of active Ras in CGNs. Isolated P7 CGNs were plated and allowed to rest for 3 hours in insulin-free medium before stimulation with Shh-N conditioned medium for the indicated durations. Lysates were collected and processed for active Ras pull-down and immunoblotting for Ras isoforms. Quantification from 3 independent experiments shows the fold change in active Ras levels compared to non-stimulated controls (red dotted line). *P* values derived from an unpaired one-tail student's t-test comparing active Ras levels between stimulated and non-stimulated cells . Error bars represents mean ± SD.

Supplementary Figure 4

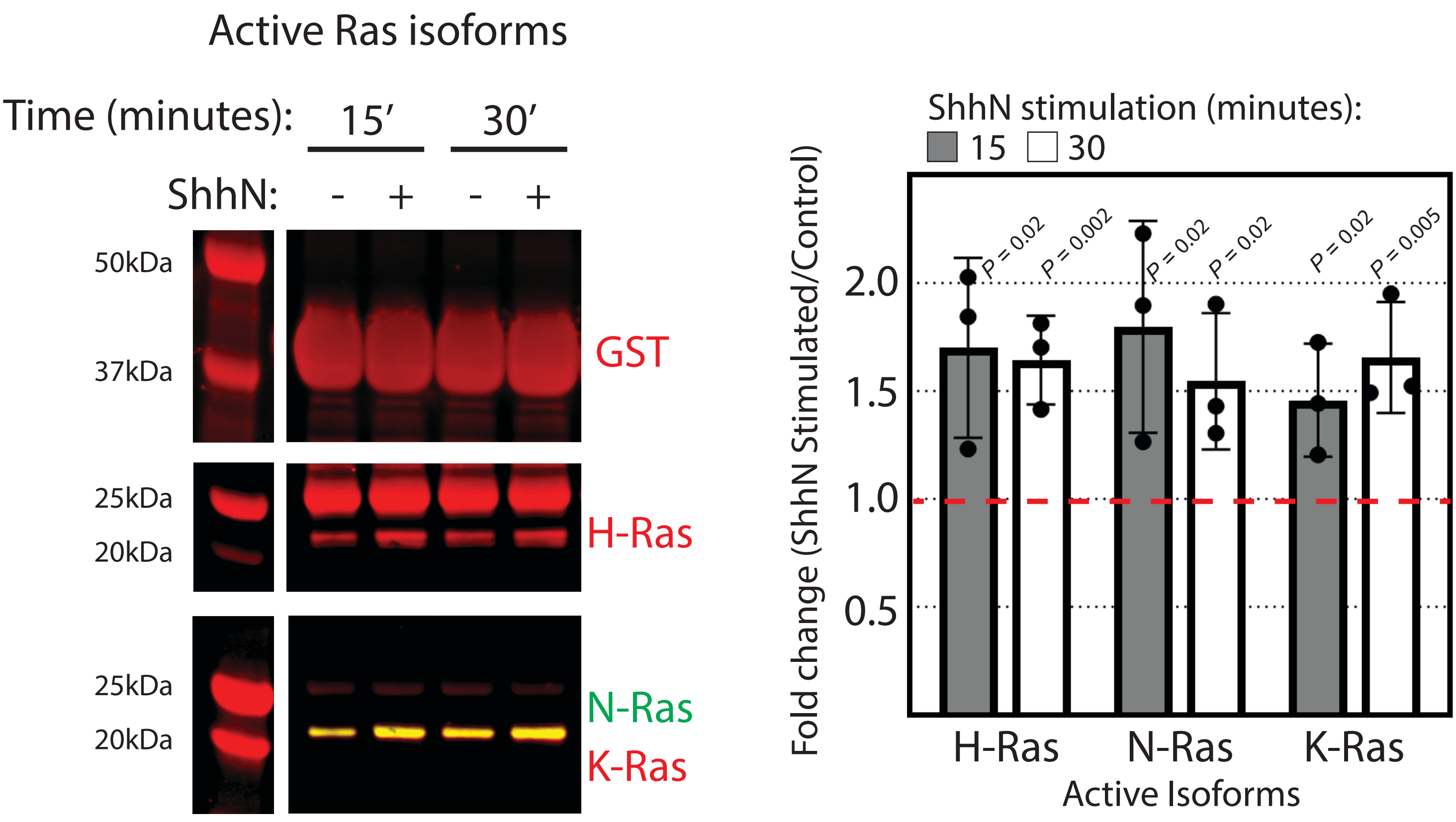

Supplementary figure 5. Shh signaling promotes primary ciliogenesis in GNPs. Isolated P7 CGNs were cultured in the presence or absence of SAG (100nM) for 48 h. Cells were fixed, and immunofluorescence staining for Arl13b was performed. Quantification of Control, n = 690; SAG, n = 566; individual cells from 3 independent experiments shows the mean percentage of ciliated cells determined by manual scoring. *P* value derived from an unpaired one-tail student's t-test. Scale bar = 10μm. Error bars represents mean ± SD.

Supplementary Figure 5

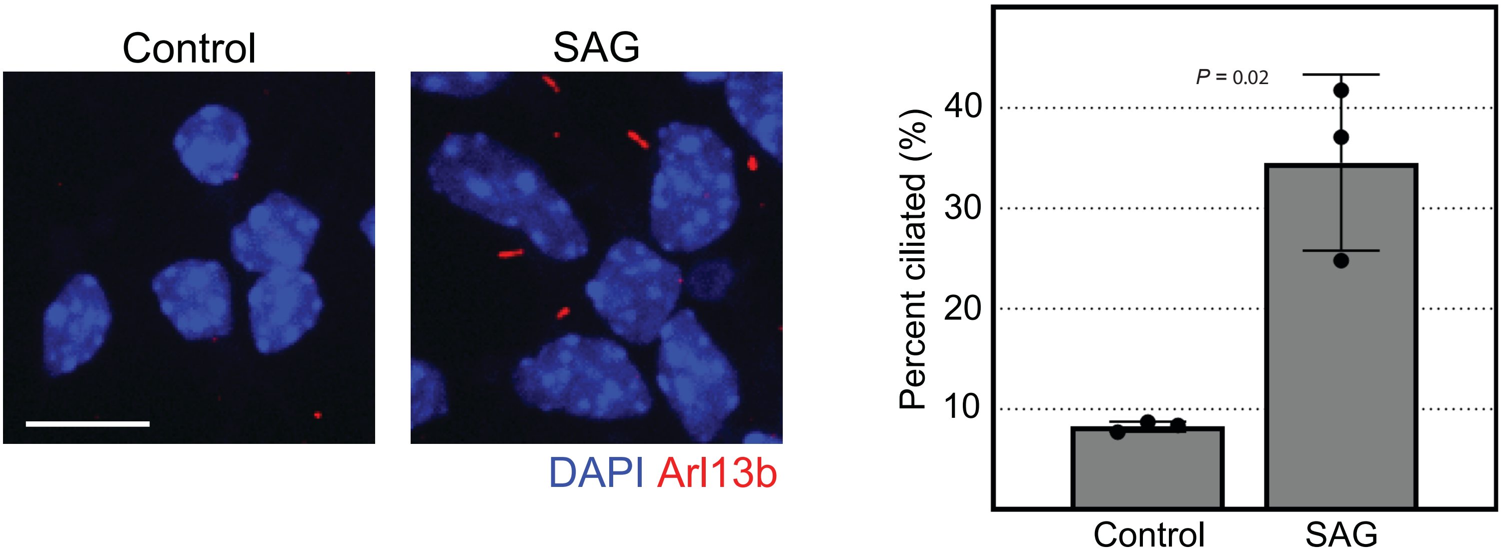

Supplementary figure 6. Knockdown of IFT88 promotes cell-cycle and early GZ exit. *Ex vivo* cerebellar pulse chase assay assessing the effects of IFT88 knockdown on GZ exit in P7 cerebella. Experimental schema is as shown. Brain slices were fixed at 24 hours for EdU staining followed by imaging and analyses. Representative images of the migration patterns with the indicated manipulations are as shown. Quantification n > 7000 individual cells in each condition from 4 independent experiments shows the migration distribution (top panel), average migration distance (middle panel), and mean proliferative index (lower panel) analyses. Horizontal length of representative images represents radial distance of 300μm from cerebella pial. *P* values derived from an unpaired one-tail student's t-test. Error bars represents mean ± SD.

Supplementary Figure 6

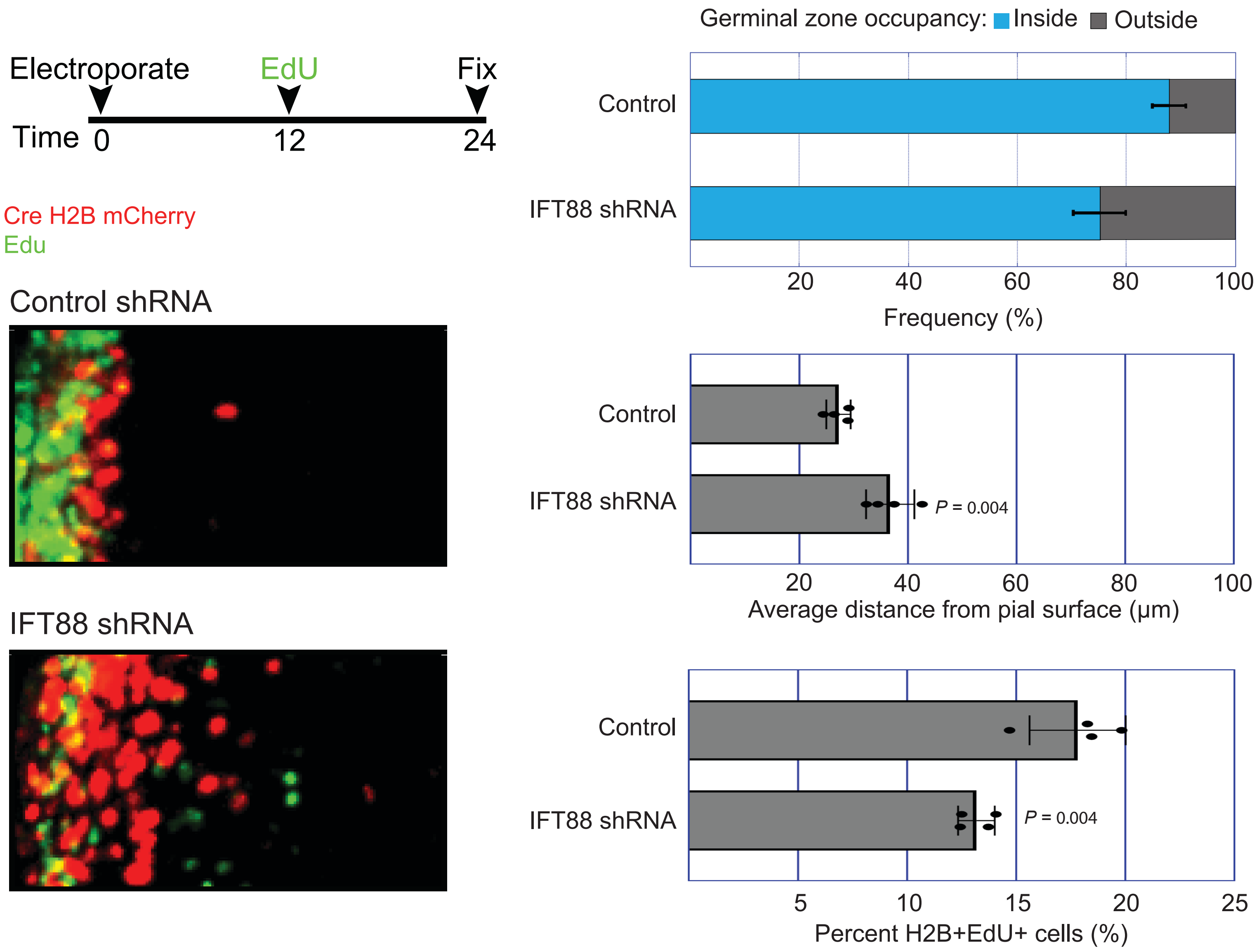

Supplementary figure 7. Shh-driven primary ciliogenesis requires Ras, Erk1/2, and Siah2. Isolated P7 CGNs were nucleofected with the indicated shRNAs and cultured for 48 hours on matrigel coated glass in the presence or absence of recombinant Shh-N (3 µg/mL). The cells were then fixed for immunofluorescence staining of followed by imaging on a spinning-disk confocal microscope. Quantification n > 800 cells in each condition from 3 independent experiments shows the mean percentage of nucleofected cells that were ciliated, as determined by manual scoring. *P* values derived from an unpaired one-tail student's t-test comparing experimental groups to "Control shRNA + Shh-N". Scale bar = 10µm. Error bars represents mean ± SD.

Supplementary Figure 7

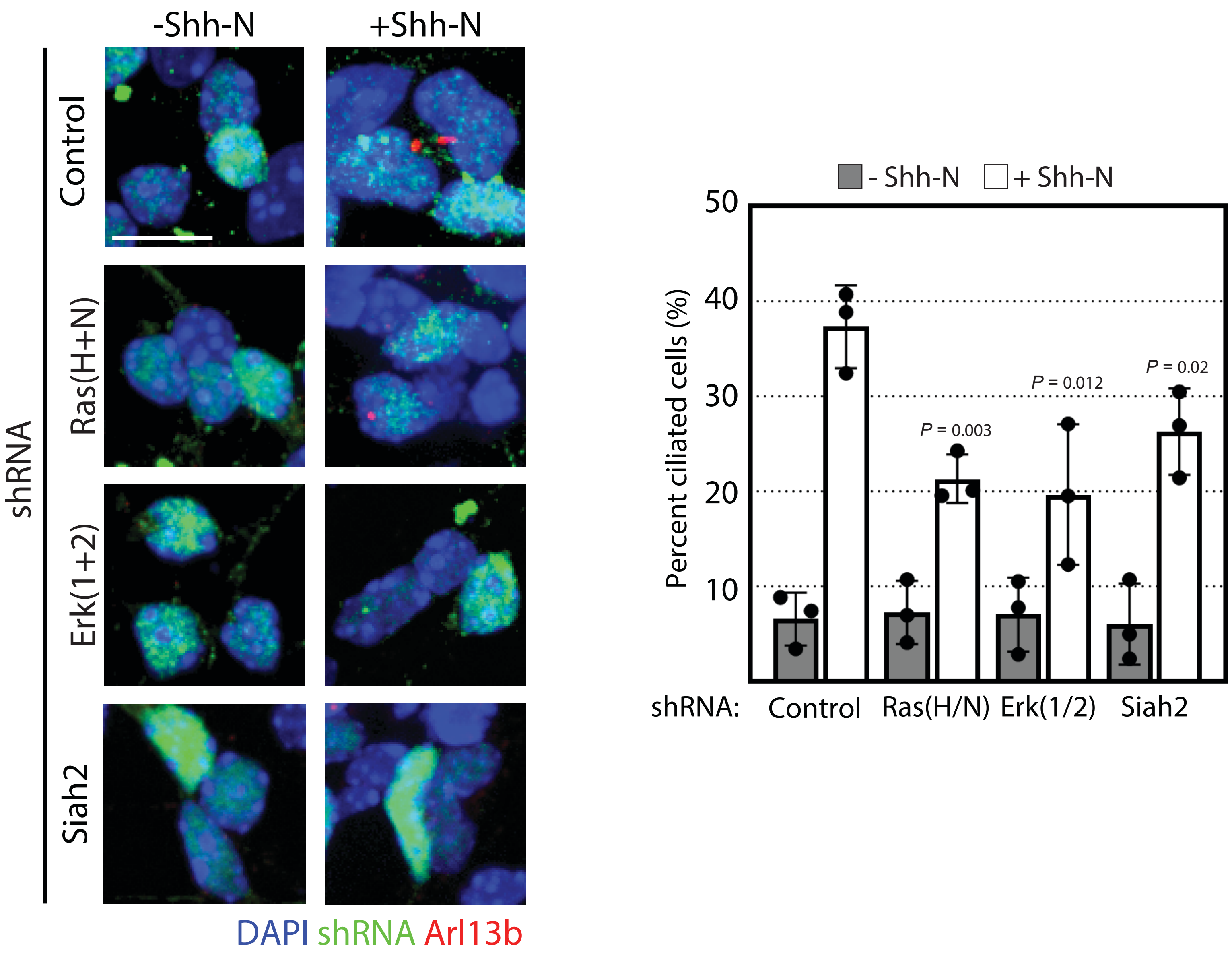

Supplementary figure 8. Siah2 controls Shh signaling by regulating primary ciliogenesis. (A) Isolated P7 CGNs were co-nucleofected with the indicated constructs and a bicistronic vector encoding Venus-tagged Arl13b and mCherry-tagged histone H2B. The cells were cultured on matrigel coated glass. Live-cell imaging with a spinning-disk confocal microscope was performed. Quantificaton n > 1300 cells in each condition from 3 independent experiments shows the mean percentage of nucleofected cells (H2B mCherry+) that were ciliated, determined by manual scoring. *P* values derived from an unpaired one-tail student's t-test comparing experimental groups to "Siah2". Scale bar = 10μm. Error bars represents mean ± SD.

Supplementary Figure 8

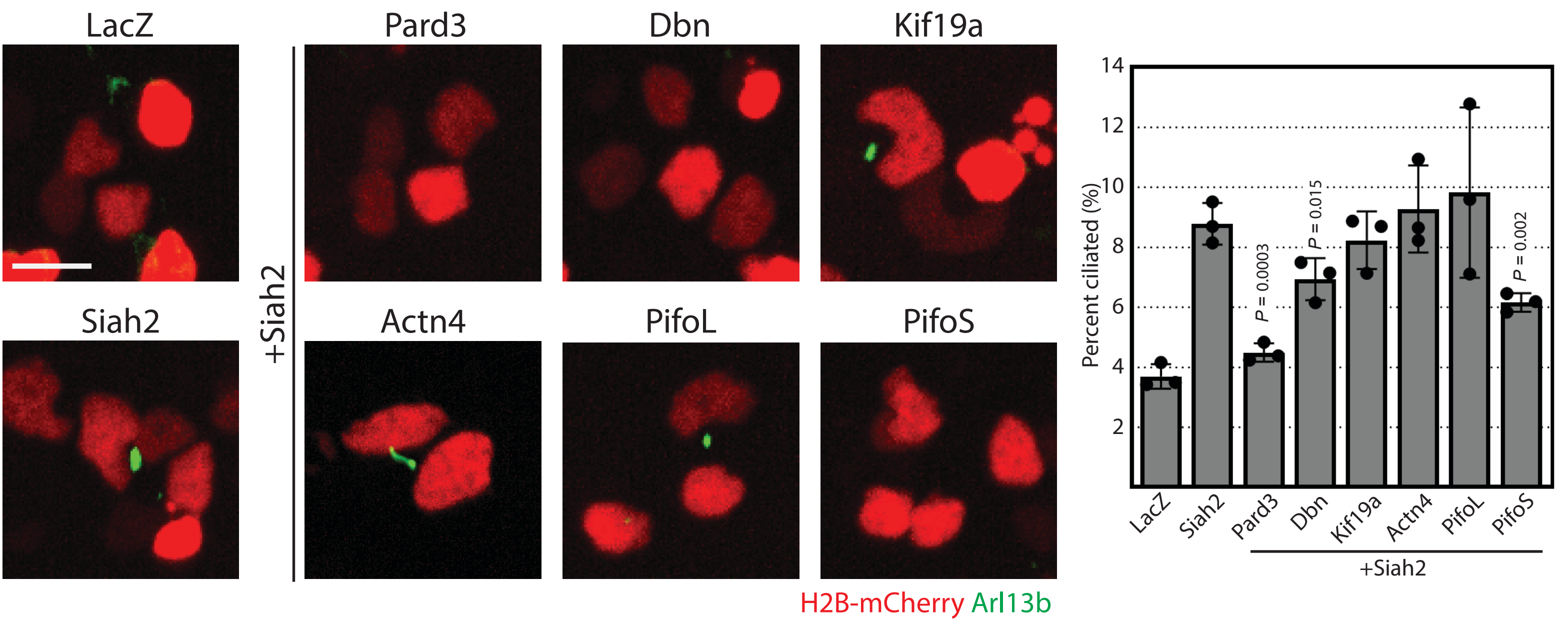

Supplementary figure 9. Igf2 from cerebellar pial have neglibible effects on primary ciliogenesis. (A) Results of *in situ* hybridization analysis of IGF2 mRNA in a P7 cerebellum. Data were obtained from the Brain Transcriptome Database (BrainTx). (**B**) Isolated P7 CGNs were plated on laminin coated glass and cultured in the presence of Igf2 (200ng/mL) or Shh-N conditioned medium (cm) for 48 hours. Cells were fixed for immunofluorescent staining of Arl13b. Quantification Igf2, n = 1385; Shh-N, n = 1538; cells from 4 independent experiments shows the mean percent ciliated cells by manual scoring. *P* value derived from an unpaired one-tail student's t-test. Scale bar = 10µm. Error bars represents mean ± SD.

Supplementary Figure 9

A

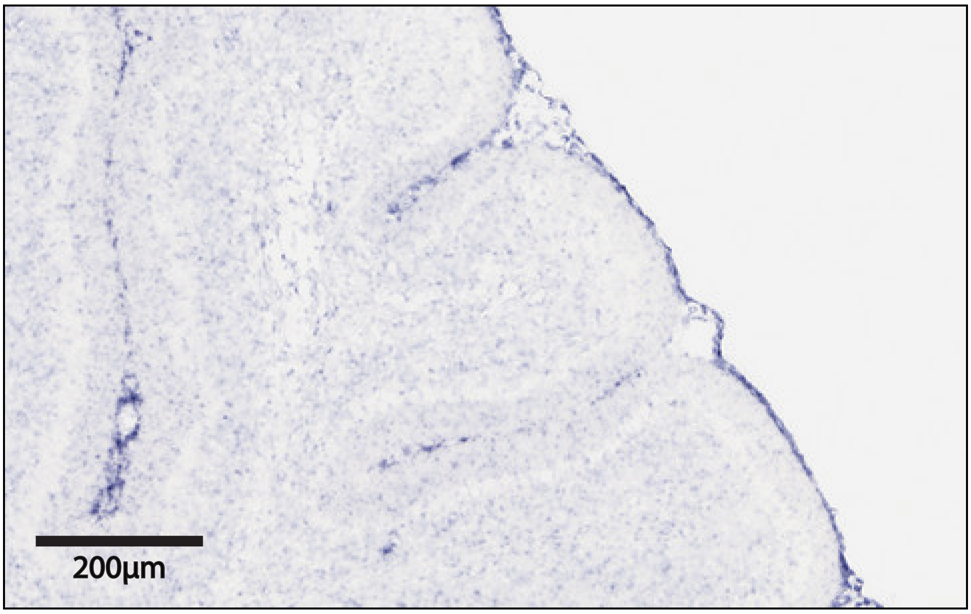

B

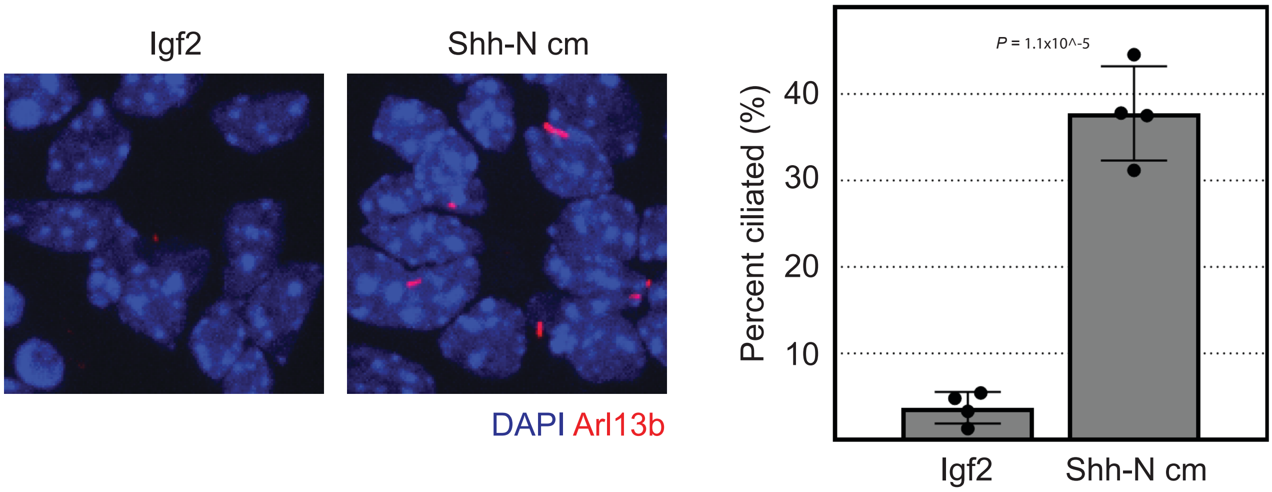

Supplementary figure 10. Laminin and vitronectin have opposing effects on primary ciliogenesis and Siah2. (**A**) Immunofluorescent staining of Vitronectin in P7 cerebellar cryosection. (**B**) Isolated P7 CGNs were nucleofected with contructs encoding integrin β1 V7373N, an auto-clustering integrin β1 mutant or RasV12T35S (Ras-Raf), a constitutively active Ras mutant that only activates Raf/Mapk and cultured on laminin coated glass for 48 hours. Cells were fixed for immunofluorescent staining of Siah2. Quantification from 3 independent experiments (n = 27 independent confocal image fields in each condition) shows the mean percent Siah2 positive cells using Slidebook. (**C** and **D**) Isolated P7 CGNs were plated on laminin or vitronectin coated glass and stimulated with control or Shh-N conditioned medium for 48 hours. Cells were fixed followed by immunofluorescence staining of (**C**) Siah2 and (**D**) Arl13b. Quantification n > 1500 cells in each condition from 3 independent experiments of mean percent Siah2 positive analysis using Slidebook or mean percent primary ciliated cells by manual scoring are shown. *P* values derived from an unpaired one-tail student's t-test comparing – (**A**) experimental groups to "LacZ"; or as indicated. n.s. = not significant. Scale bar = 10µm. Error bars represents mean ± SD.

Supplementary Figure 10

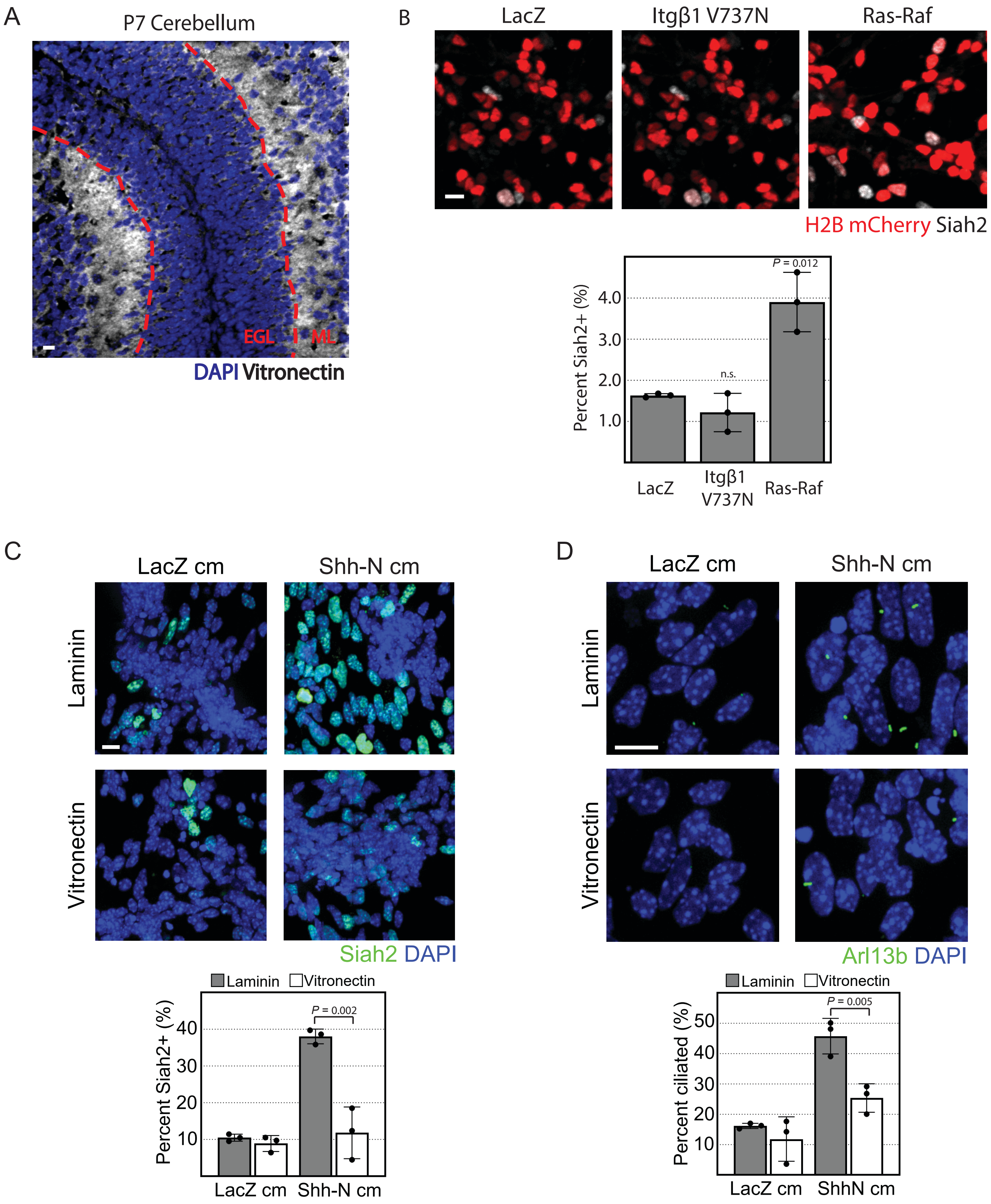

Supplementary figure 11. Integrin β1 and Ras are required to maintain expression of Siah2. (**A**) Isolated CGNs from P7 *Integrin* $\beta 1^{F/F}$ cerebella were nucleofected with active or inactive mutants of Cre and plated laminin. The cells were stimulated with control or Shh-N conditioned medium (cm) for 48 hours followed by fixation and immunofluorescence staining for Siah2. Quantification n = 27 independent confocal image fields in each condition from 3 independent experiments, shows mean percent Siah2 positive analysis using Slidebook. (**B**) Isolated P7 CGNs from wildtype cerebella was plated on laminin coated glass and stimulated with Shh-N cm with or without addition of Farnesylthiosalicylic acid (FTA) for 48 hours. Cell were fixed follow by immunofluorescent staining for Siah2. Quantification n = 27 independent confocal image fields in each condition from 3 independent experiments, shows mean percent Siah2 positive analysis using SlideBook. *P* values derived from an unpaired one-tail student's t-test. Scale bar = 10μm. Error bars represents mean ± SD. (**C**) Full scans of blots from **Figure 6F.** Dotted-lined box show cropped regions.

Supplementary Figure 11

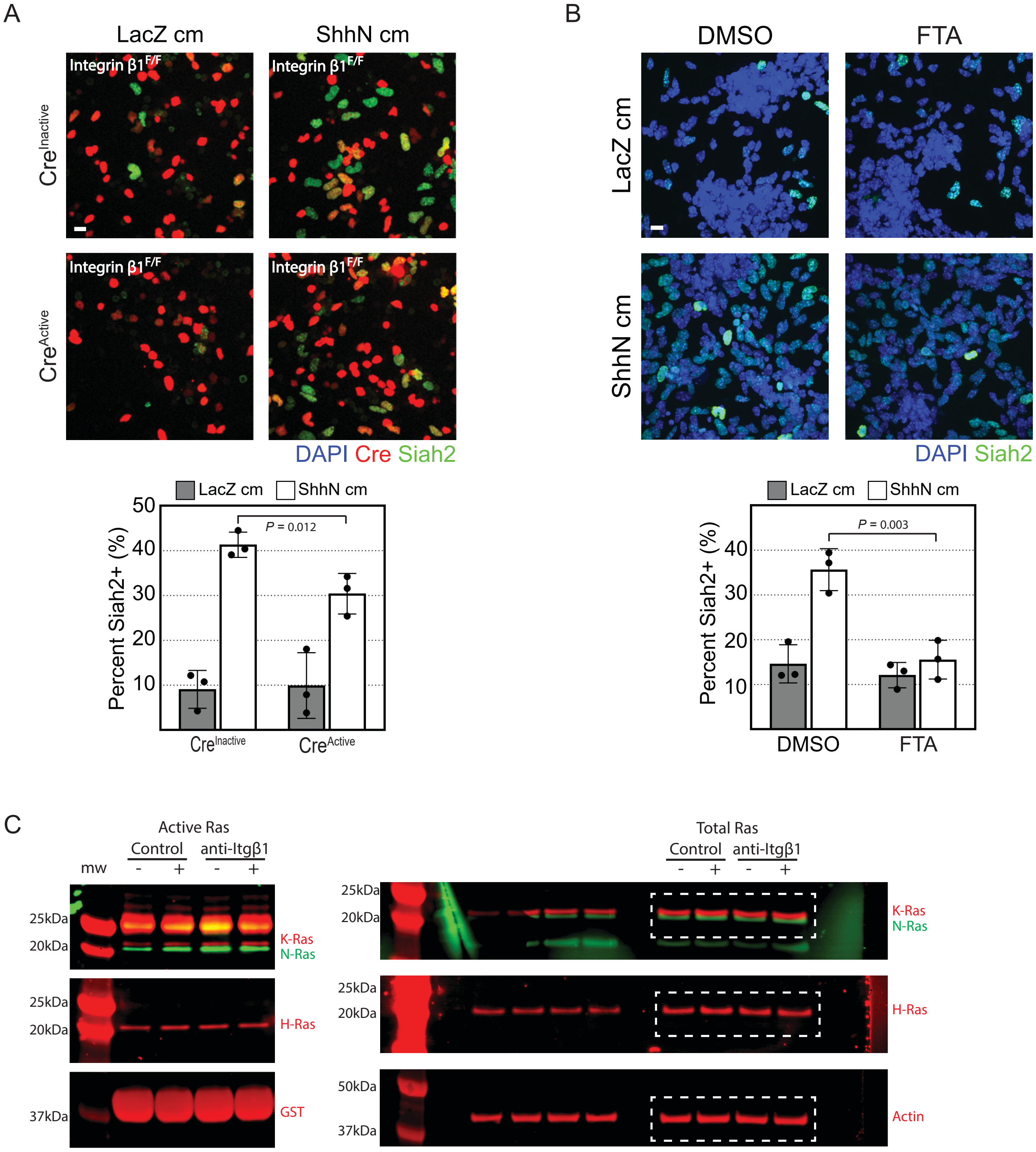

Supplementary figure 12. Activation of Itgβ1-Ras and Shh have no synergistic effect on GZ exit. *Ex vivo* cerebellar pulse-chase experiment on P7 wildtype cerebella examining the effects of integrin β1 sensitization or Ras co-activation with Shh signaling on GZ exit. Quantification of 3 independent experiments, for at least n > 8000 individual cells counted for every condition, show the mean frequency distribution within and outside the EGL (top panel) and the average migration distance (lower panel). Horizontal length of representative images represents a radial distance of 300μm from the cerebella pial. *P* value derived from an unpaired one-tail student's t-test when compared to Group 1. n.s. = not significant. Error bars represents mean ± SD.

Supplementary Figure 12

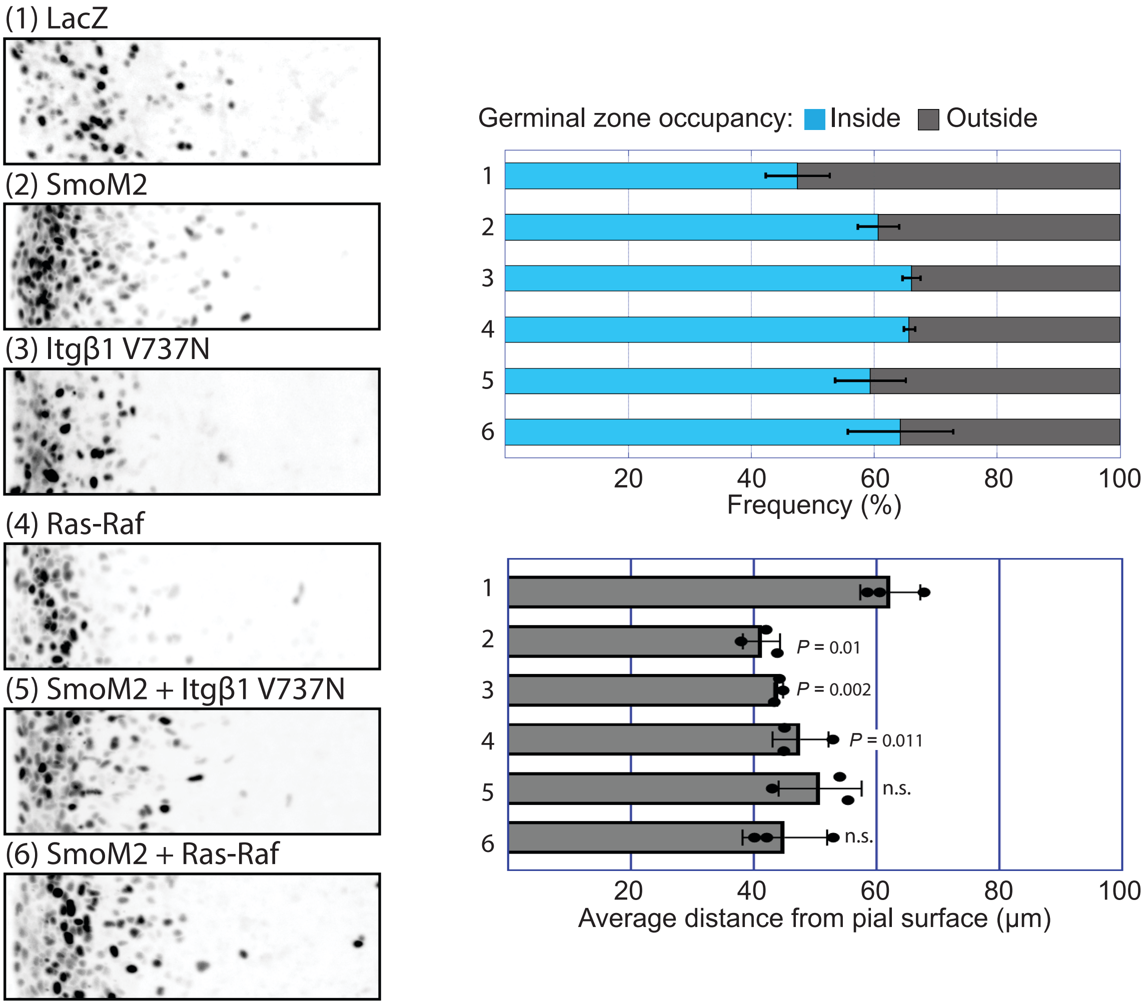

Supplement: Supplementary file 1 — Supplementary Information [file 41467_2020_19063_MOESM1_ESM.pdf]
